# Mortality and microbial diversity after allogeneic hematopoietic stem cell transplantation: secondary analysis of a randomized nutritional intervention trial

**DOI:** 10.1038/s41598-021-90976-z

**Published:** 2021-06-02

**Authors:** Kristin J. Skaarud, Johannes R. Hov, Simen H. Hansen, Martin Kummen, Jørgen Valeur, Ingebjørg Seljeflot, Asta Bye, Vemund Paulsen, Knut E. A. Lundin, Marius Trøseid, Geir E. Tjønnfjord, Per Ole Iversen

**Affiliations:** 1 Department of Haematology, Oslo University Hospital, P.O. Box 4950 Nydalen, 0424 Oslo, Norway; 2 Department of Nutrition, Institute of Basic Medical Sciences, University of Oslo, Oslo, Norway; 3 Section of Gastroenterology, Department of Transplantation Medicine, Oslo University Hospital, Oslo, Norway; 4 Institute of Clinical Medicine, University of Oslo, Oslo, Norway; 5 Norwegian PSC Research Center and Research Institute of Internal Medicine, Division of Surgery, Inflammatory Diseases and Transplantation, Oslo University Hospital, Oslo, Norway; 6 Department of Gastroenterology, Oslo University Hospital, Oslo, Norway; 7 Unger-Vetlesen Institute, Lovisenberg Diaconal Hospital, Oslo, Norway; 8 Center for Clinical Heart Research, Department of Cardiology, Oslo University Hospital Ullevål, Oslo, Norway; 9 Regional Advisory Unit for Palliative Care, Department of Oncology, Oslo University Hospital, Oslo, Norway; 10 Department of Nursing and Health Promotion, Faculty of Health Sciences, Oslo Metropolitan University, Oslo, Norway; 11 K.G. Jebsen Centre for Coeliac Disease Research, University of Oslo, Oslo, Norway; 12 Section of Clinical Immunology and Infectious Diseases, Oslo University Hospital, Oslo, Norway; 13 K.G. Jebsen Centre for B Cell Malignancies, University of Oslo, Oslo, Norway

**Keywords:** Medical research, Biomarkers

## Abstract

Gut mucosal barrier injury is common following allogeneic hematopoietic stem cell transplantation (allo-HSCT) and associated with poor clinical outcomes. Diet is critical for microbial diversity, but whether nutritional support affects microbiota and outcome after allo-HSCT is unknown. We present a secondary analysis of a randomized controlled nutritional intervention trial during allo-HSCT. We investigated if the intervention influenced gut microbiota, short-chain fatty acids (SCFAs), and markers of gut barrier functions, and if these parameters were associated with clinical outcomes. Fecal specimens were available from 47 recipients, and subjected to 16S rRNA gene sequencing. We found no significant differences between the intervention group and controls in investigated parameters. We observed a major depletion of microbiota, SCFAs, and altered markers of gut barrier function from baseline to 3 weeks post-transplant. One-year mortality was significantly higher in patients with lower diversity at 3 weeks post-HSCT, but not related to diversity at baseline. The relative abundance of *Blautia* genus at 3 weeks was higher in survivors. Fecal propionic acid was associated with survival. Markers of gut barrier functions were less strongly associated with clinical outcomes. Possibly, other strategies than dietary intervention are needed to prevent negative effects of gut microbiota and clinical outcomes after allo-HSCT.

ClinicalTrials.gov (NCT01181076).

## Introduction

The toxicity of allogeneic hematopoietic stem cell transplantation (allo-HSCT) and extended exposure to systemic antibiotics cause mucosal barrier injury, leading to increased intestinal permeability and altered microbiota^1^. Mucosal barrier injury and bacterial translocation have been associated with acute graft-versus-host-disease (aGVHD) and survival^2–4^. In line with this, markers of mucosal barrier injury have identified patients at risk of gastrointestinal toxicity and aGVHD in observational studies^2–4^. High diversity of the gut microbiota, both before allo-HSCT and until engraftment, is associated with reduced aGVHD and improved overall survival^5,6^. Moreover, specific bacteria have been associated with allo-HSCT outcomes. For example, *Blautia* has been associated with reduced mortality from aGVHD whereas *Enterococcus has been* linked to reduced overall survival^7,8^.

Diet is a major determinant of gut microbiota composition^9^. Specifically, dietary fibers are known to interact with gut microbes and lead to higher production of short-chain fatty acids (SCFAs), which are important energy source for the intestinal epithelium^10^. Depletion of SCFAs has been associated with aGVHD in children^11^ and in animal models^12,13^. In line with this, SCFAs have been associated with protection from chronic GVHD^14^. However, the role of diet for microbial composition and functioning in allo-HSCT patients is unclear and complicated by the fact that these patients do not eat conventional diets. Their nutritional intake varies between normal cooked food, oral nutritional supplements, tube feeding, and parenteral nutrition^15,16^. One study reported prompt post-HSCT recovery of gut microbiota and SCFAs in children receiving tube feeding compared to parenteral feeding, but with no impact of clinical outcomes^17^. Studies of markers of gut barrier functions in relation to nutritional support of HSCT are lacking. In a randomized controlled trial (RCT) we found no effect of optimized energy and protein intake preferably via the enteral route on global quality of life, oral mucositis, and aGVHD three months post-HSCT^18^. Moreover, the nutrition intervention had no impact on one-year survival^19^. The aim of the current study was to use data from this RCT to assess: (i) the impact of the nutritional intervention on microbiota, fecal SCFAs, and biomarkers of gut barrier function, and (ii) the impact of microbiota, SCFAs, and biomarkers of gut barrier functions on survival and aGVHD.

## Materials and methods

### Patients

Our open, two-armed randomized controlled trial (RCT) has been detailed previously^18^. Briefly, we included 117 patients ≥ 18 years of age undergoing allo-HSCT after myeloablative conditioning for a hematological malignancy. Patients were randomly assigned using a 1:1 ratio (block sizes of 10) to receive optimized energy and protein intake preferably via the enteral route (intervention: n = 57) or standard total parenteral nutrition (TPN) (control: n = 60)^18^. Fecal samples were collected throughout the last five randomization blocks resulting in fecal samples from 47 patients at baseline and 3 weeks post-HSCT (Supplementary Information). The samples were immediately frozen at − 80 °C or immediately stored at 4 °C and frozen within 24 h at − 80 °C. Blood samples were collected from the 117 patients at baseline and 3 weeks post-HSCT, immediately frozen and stored at − 80 °C. Data were collected from August 2010 to February 2017 at Oslo University Hospital, Norway. All patients gave written informed consent before entry the study, and the study was performed in accordance with the Declaration of Helsinki. The study was approved by The Regional Committee for Medical and Health Research Ethics South East Norway (#S-09136c 2009/2115) and the Data Protection Officer, Oslo University Hospital. The RCT is registered with Clinical Trials number NCT01181076.

### Treatment procedure

The procedures have been detailed^18^. The nutritional intervention aimed at a minimum daily energy intake of 126 kJ/kg body weight and a protein intake of 1.5–2.0 g protein/kg body weight, preferably via the enteral route^18^. Patients received routine hospital food and were encouraged to consume energy-enriched and lactose-reduced snacks and oral supplements on a daily basis. A nasoenteric tube was inserted within day + 5 post-HSCT and a non-fiber, hydrolyzed medium triglyceride fat formula administered. We supplemented with parenteral nutrition (PN) if intolerance for enteral nutrition e.g. in the case of gut GVHD and diarrhea. The control group received routine practice for nutritional support, energy and protein requirements were not calculated, dietary intake not recorded, and tube feeding not administered. TPN was given at the discretion of the treating physician.

During hospitalization the intervention group received significantly more (median (range)) energy (kJ/kg) and protein (g/kg) compared to a reference group for the control group, 131.9 (58.2–178.7), 1.1 (0.5–1.5) and 99.2 (50.2–139.8), 0.6 (0.4–1.0) (*p* < 0.001, *p* < 0.001), respectively. In the intervention group a nasoenteric tube was inserted in 55 patients (two refused) and EN commenced in 49 patients (six wanted to remove the tube before commencing EN). The tubes stayed in position a median of 12 (1–50) days^18^. We observed no effect of recommended energy and protein intake on the main endpoint global quality of life or the secondary outcomes oral mucositis and aGVHD three months post-HSCT^18^. The nutritional intervention had no impact on one-year survival^19^.

Trimethoprim-sulfamethoxazole was given prophylactic. Gentamicin and penicillin were preferred upon neutropenic fever^20^ and ceftazidime or piperacillin-tazobactam and meropenem in combination with antifungal treatment as second and third line treatments, respectively. Acute GVHD prophylaxis consisted of cyclosporine and methotrexate.

### Definition of study outcomes

Sample size calculation was based on the primary endpoint of the original RCT as previously described^18^. In the current study, the outcomes were gut microbial diversity, fecal SCFAs, circulating markers of gut leakage (a proxy for gut mucosal barrier function) at baseline and 3 weeks post-HSCT, aGVHD, one-year survival, and non-relapse mortality. One year post-HSCT was defined as the day the patients arrived for the regular one year visit. Gut microbiota composition was classified according to measures of alpha diversity (observed operational taxonomic units [OTUs] and Shannon diversity index), inter-individual (beta) diversity, as well as relative abundances of microbes at the genus level. Death was defined as death from any cause one year post-HSCT. Non-relapse mortality (NRM) was defined as death from any cause except from relapse. We reported the highest grade of aGVHD^21^. Antibiotic usage was given as number of days of treatment.

### Analyses of gut microbiota, short-chain fatty acids and markers of gut barrier functions

Microbial DNA was extracted from fecal samples using PSP Spin Stool DNA Kit (Stratec Molecular GmbH) and the V3-V4 region of the 16S rRNA gene was amplified followed by sequencing on the Illumina MiSeq platform (REF PMID 24558975). Quality control and closed reference OTU mapping to the Silva database^22^ (version 128, reference OTUs pre-clustered at 97% sequence similarity) was performed using Quantitative Insights Into Microbial Ecology version 1.9.1^23^ (Supplementary Information). The samples were rarefied (sub-sampled) to an OTU count of 15,004 per sample, and all further analyses were performed on this rarefied dataset.

Fecal concentrations of SCFAs were analyzed using vacuum distillation followed by gas chromatography^24–26^. Levels of the following SCFAs were assessed: acetic acid, propionic acid, iso-butyric acid, butyric acid, iso-valeric acid, valeric acid and total SCFAs. The plasma levels of (the gut-leakage markers) intestinal fatty acid binding protein (I-FABP), lipopolysaccharide binding protein (LBP) and soluble cluster of differentiation (sCD14) were measured by ELISAs (Hycult Biotech, Uden, The Netherlands and R&D Systems Europe, Oxfordshire, UK, respectively). The inter-assay coefficients of variation were 5.8%, 9.8% and 7.4%, respectively.

### Statistical analyses

Comparison of continuous variables was performed with Mann–Whitney U test. We used Wilcoxon signed rank test for comparisons between data obtained at baseline and 3 weeks after transplantation. For analysis of bacterial abundances only genera present in at least 50% of the individuals in at least one of the groups analyzed were included. The ability of alpha diversity measures, SCFAs and markers of gut barrier functions to patients reaching an endpoint (death of all causes or NRM) were analyzed by comparing Receiver Operating Characteristic (ROC) curves. The optimal cut-off was determined using Youden’s index, and when the AUC was significant, Kaplan–Meier plots were used to compare the time to death of any cause or NRM, comparing values above versus below the cut-off using log rank test. Correlation analysis was performed using Spearman’s test. Acute GVHD was categorized as “yes” or “no”. We used the Hematopoietic Cell Transplantation-specific comorbidity index (HCTI-CI) to classify the patients into low and intermediate/high risk categories. Due to few events we did not analyze associations between SCFAs at 3 weeks and death, NRM, aGVHD and HCTI-CI. Multivariate analysis of beta diversity between the intervention and control groups at baseline and at 3 weeks and changes from baseline to 3 weeks was performed with a permutational multivariate analysis of variance (PERMANOVA). Due to multiple comparisons, false discovery rates were also calculated for the comparisons of bacterial taxa, according to the Benjamini–Hochberg method and denoted Q_FDR_. A *p* < 0.05 was considered statistically significant. Analyses were performed using IBM-SPSS 26 (IBM Corp., Armonk, NY). R package, version 3.6.1^27^ and MedCalc (MedCalc Software bvba, Ostend, Belgium).

## Results

Characteristics at baseline of the 47 patients (intervention: n = 23, control: n = 24) with fecal samples are shown in Table 1, and were similar to the 117 (intervention: n = 57, control: n = 60) of the original trial cohort (Supplementary Table S2)^18^. In total 12 (26%) patients died during one year follow-up (6 in the intervention and 6 in the control group). One-year overall survival was similar in the intervention and control group (*[* = 0.850) (Supplementary, Fig. S1). NRM was 5 (11%) (3 in the intervention group and 2 controls). Nine (19%) suffered a relapse (5 in the intervention group and 4 controls). Two patients who suffered a relapse were still alive at one year. A total of 36 (77%) patients (18 in both study groups) had aGVHD grade 0–1 and a total of 11 grade 2–4 (5 in the intervention group and 6 controls). Median (range) days of systemic antibiotic therapy was 15 (8–22) and 16 (8–31) in the intervention and control group, respectively.

**Table 1:** Clinical characteristics at baseline of the two study groups.

	Intervention (n = 23)	Control (n = 24)
Age (years)	45 (19–63)	35 (21–54)
Female	9 (39.1)	13 (54.2)
**Underlying disease**		
AML	15 (65)	15 (62)
ALL	1 (4)	3 (13)
CML	–	3 (13)
CMML	2 (9)	–
aCML	1 (4)	–
MDS	4 (18)	1 (4)
ABL	–	1 (4)
MCL	–	1 (4)
**Donor**		
HLA − identical sibling	4 (17)	5 (21)
HLA − identical unrelated	19 (83)	19 (79)
**Stem-cell source**		
Bone marrow	17 (74)	19 (79)
Peripheral-blood hematopoietic cells	6 (26)	5 (21)
Sex mismatch^a^	8 (35)	–
**Conditioning**		
Busulfan + cyclophosphamide	23 (100)	22 (92)
Total body irradiation + cyclophosphamide	–	2 (8)
**HCTI** − **CI risk groups**		
Low risk	15 (66)	18 (75)
Intermediate risk	4 (17)	5 (21)
High risk	4 (17)	1 (4)
**EBMT score**		
0–3	17 (74)	19 (79)
4	5 (22)	3 (13)
5–7	1 (4)	2 (8)
**Performance status ECOG**		
0	23 (100)	24 (100)
**BMI**		
Underweight	–	4 (17)
Normal weight	15 (65)	11 (46)
Overweight	7 (31)	8 (33)
Moderately obese	–	1 (4)
Severely obese	1 (4)	–

Values are numbers (%) or median (range). AML Acute myeloid leukemia, ALL Acute lymphocytic leukemia, CML Chronic myeloid leukemia, CMML Chronic myelomonocytic leukemia, aCML atypical Chronic myeloid leukemia, MDS Myelodysplastic syndrome, ABL Acute basophilic leukemia, MCL Mast cell leukemia, HCTI-CI Hematopoietic Cell Transplantation-specific comorbidity index, EBMT score European Group for Blood and Marrow Transplantation score, ECOG Eastern cooperative oncology group, BMI Body mass index.

^a^Sex mismatch was defined as female donor-to-male recipient.

### Impact of the nutritional intervention and allo-HSCT on microbial diversity, SCFAs and markers of gut barrier functions

**Figure 1:**
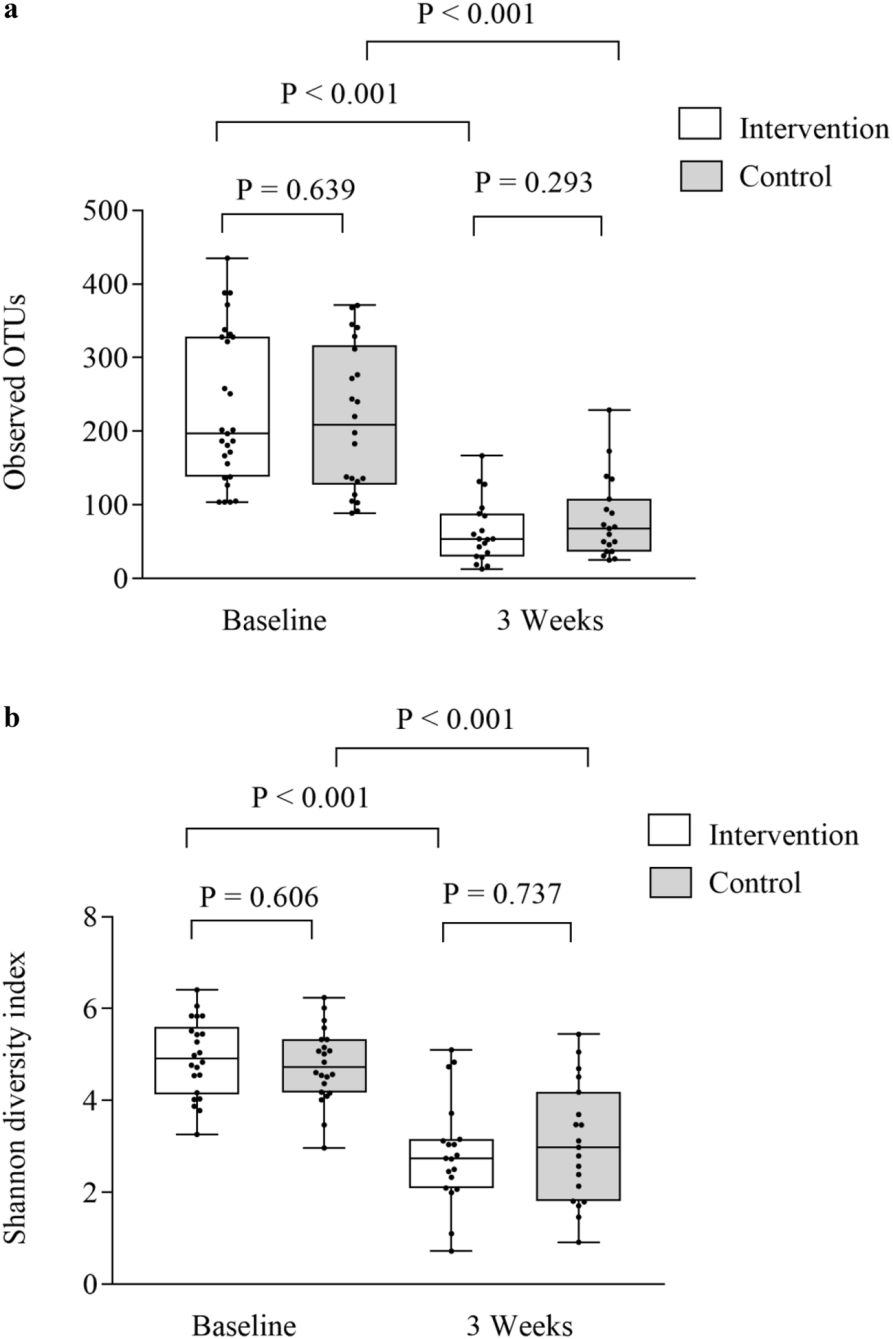
Gut microbial diversity and the intervention and control group at baseline and 3 weeks. (**a**) Observed operational taxonomic units (OTUs) and groups, (**b**) Shannon diversity index and groups. Data are observed operational taxonomic units (OTUs) and Shannon diversity index given as individual values (dots) and box plots (median and interquartile range) with minimum and maximum values.

**Figure 2:**
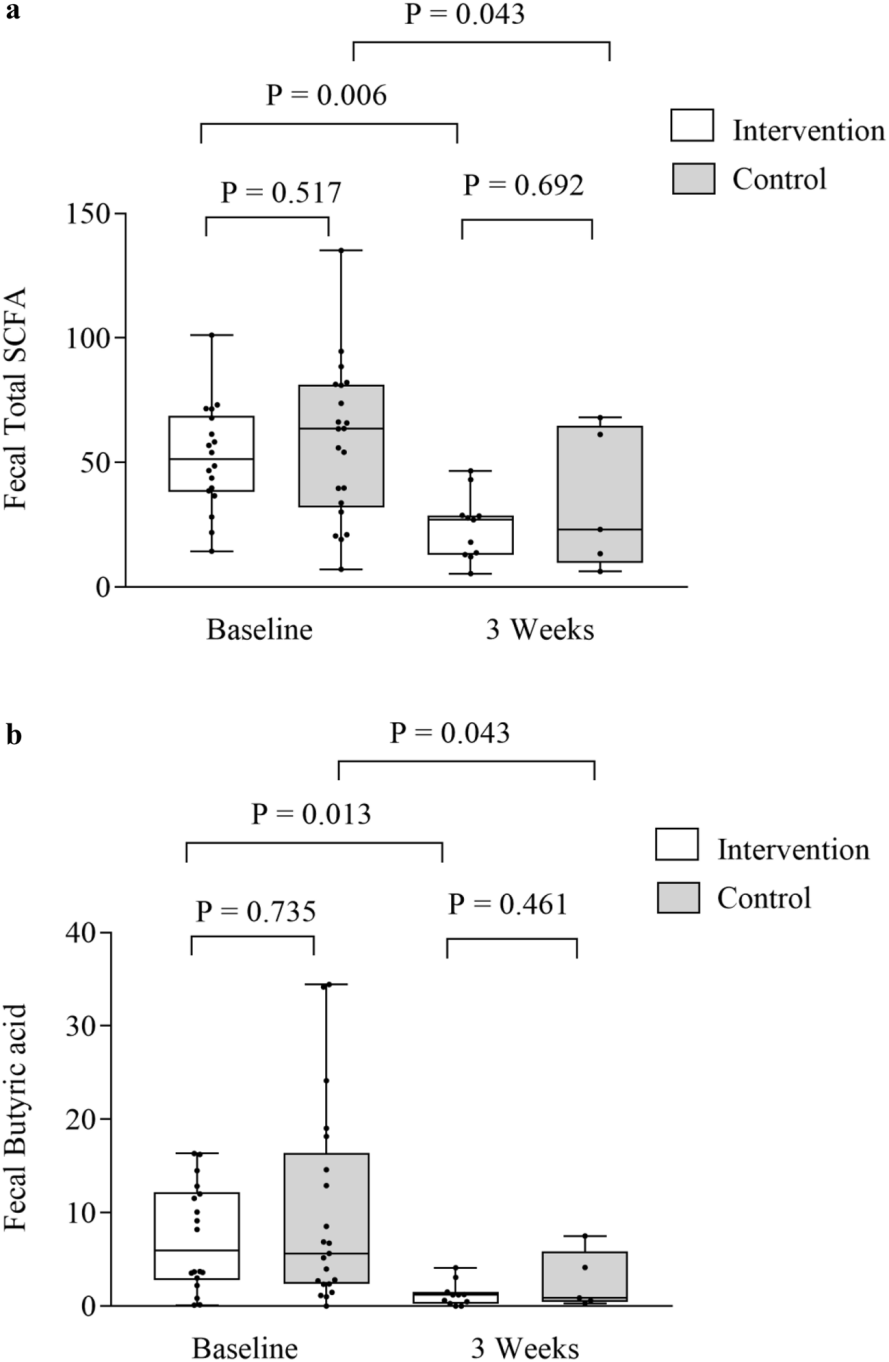
Fecal total SCFAs and the intervention and control group at baseline and 3 weeks. Data are total SCFAs given as individual values (dots) and box plots (median and interquartile range) with minimum and maximum values.

**Figure 3:**
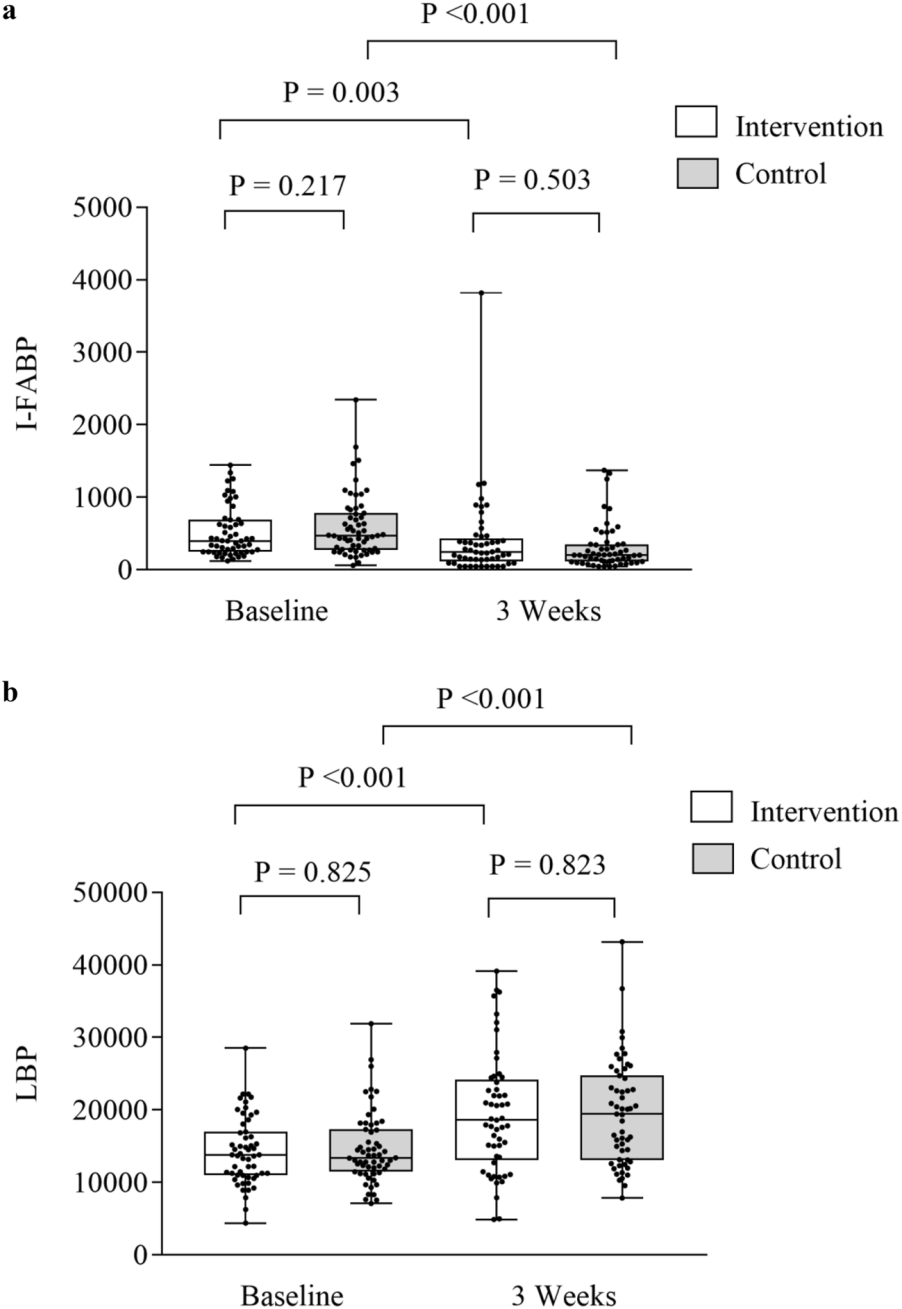
Markers of gut barrier functions and the intervention and control group at baseline and 3 weeks. (**a**) Intestinal fatty acid binding protein (I-FABP) and groups, (**b**) lipopolysaccharide binding protein (LBP) and groups. Data are I-FABP and LBP given as individual values (dots) and box plots (median and interquartile range) with minimum and maximum values.

Significant reductions were found for observed OTUs and Shannon diversity index in both the intervention and control group from baseline to 3 weeks (*p* < 0.001 for all), but with no differences between the groups (0.293 ≤ *p* ≤ 0.737), as shown in Fig. 1a, b and Supplementary Tables S2, S3. The relative abundance of the *Blautia* genus dropped in the intervention group, but not for the controls from baseline to 3 weeks (*p* = 0.048 and *p* = 0.113, respectively). There were no differences between the two study groups in the relative abundance of *Blautia* genus at 3 weeks (*p* = 0.316). An increase in relative abundance of *Enterococcus* was observed in both groups (0.001 ≤ *p* ≤ 0.021), but with no differences between the groups (*p* = 0.097, Supplementary Tables S3, S4). Also, the beta diversity dropped in both groups from baseline to 3 weeks (PERMANOVA pseudo-F = 3.64, *p* = 0.002 and pseudo-F = 4.38, *p* = 0.001), but with no differences between the groups (PERMANOVA pseudo-F = 0.78, *p* = 0.661 and pseudo-F = 1.16, *p* = 0.292, respectively). Significant reductions were observed for most SCFAs in both the intervention and control group from baseline to three weeks (0.006 ≤ *p* ≤ 0.500), but with no difference between the groups (Fig. 2a, b, Supplementary Tables S5, S6). Also, reductions were observed for I-FABP and increases of LBP in both groups from baseline to 3 weeks (< 0.001 ≤ *p* ≤ 0.003), but with no difference between them (0.503 ≤ *p* ≤ 0.823) as shown in Fig. 3a, b and Supplementary Tables S7, S8. No significant changes were observed for sCD14 between the groups and the two time points (Supplementary Tables S7, S8). Results of analysis of correlation between alpha diversity, SCFAs and gut leakage markers after pooling the two groups at the two time points are shown in Supplementary Tables 9, 10. We found minor correlations between alpha diversity, *Blautia* abundance, SCFAs and markers of gut barrier functions at baseline (− 0.03 ≤ r ≤ 0.41) (0.016 ≤ P ≤ 0.895). At 3 weeks, correlations were found for alpha diversity, *Blautia* abundance and specific SCFAs (− 0.02 ≤ r ≤ 0.66 and 0.011 ≤ *p* ≤ 0.683). Weaker correlations were observed for microbiota and specific gut leakage markers at 3 weeks (− 0.07 ≤ r ≤ 0.45 and 0.005 ≤ *p* ≤ 0.659, Supplementary Tables S9, S10).

### Impact of microbial diversity on clinical outcomes

**Figure 4:**
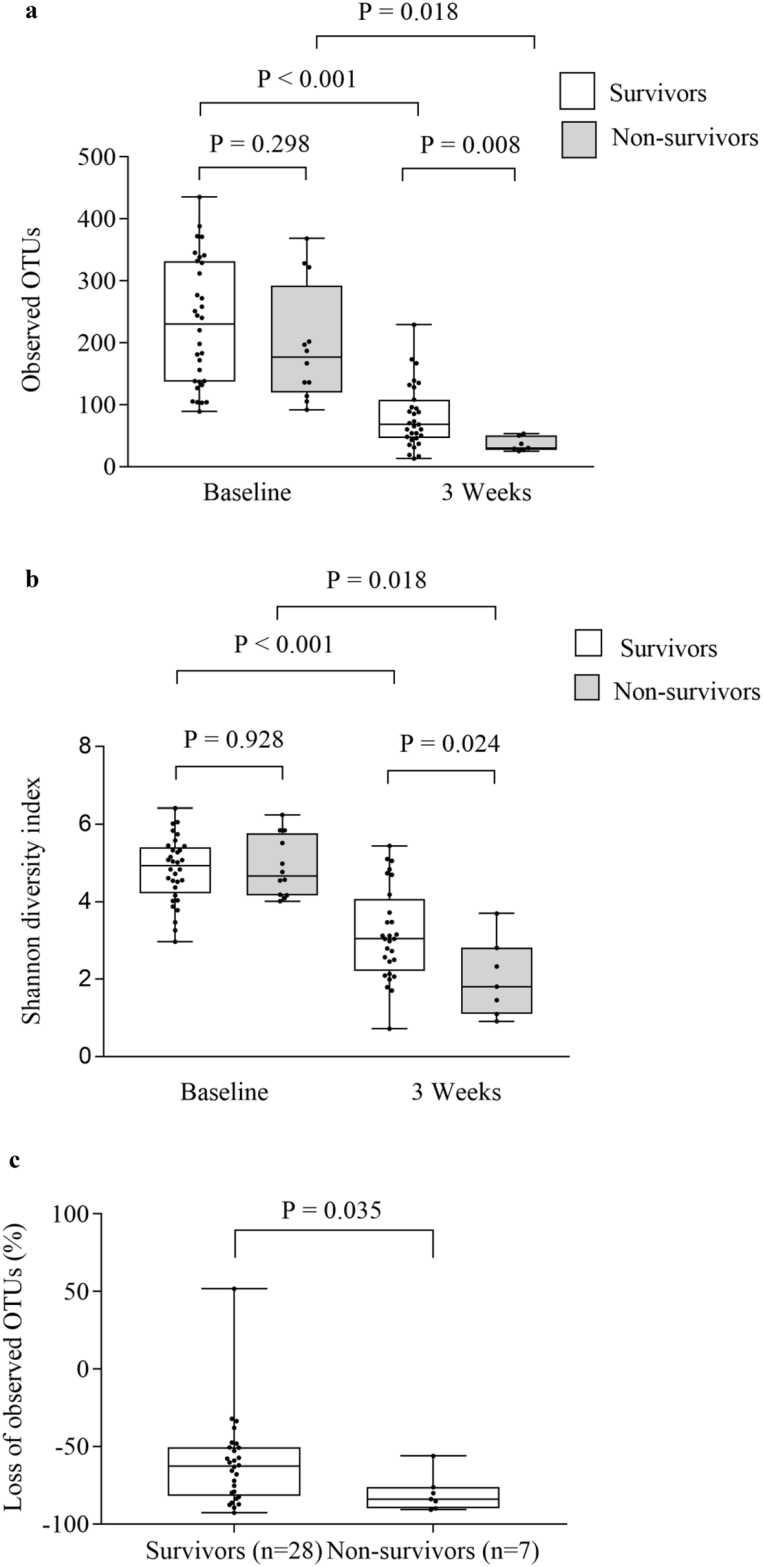
Gut microbiota diversity and one-year survival. (**a**) Observed operational taxonomic units (OTUs) at baseline and 3 weeks and one-year survival, (**b**) Shannon diversity index at baseline and 3 weeks and one-year survival, (**c**) Loss of observed OTUs from baseline to 3 weeks and one-year survival. Data are observed OTUs and Shannon diversity index, and given as individual values (dots) and box plots (median and interquartile range) with minimum and maximum values.

**Figure 5:**
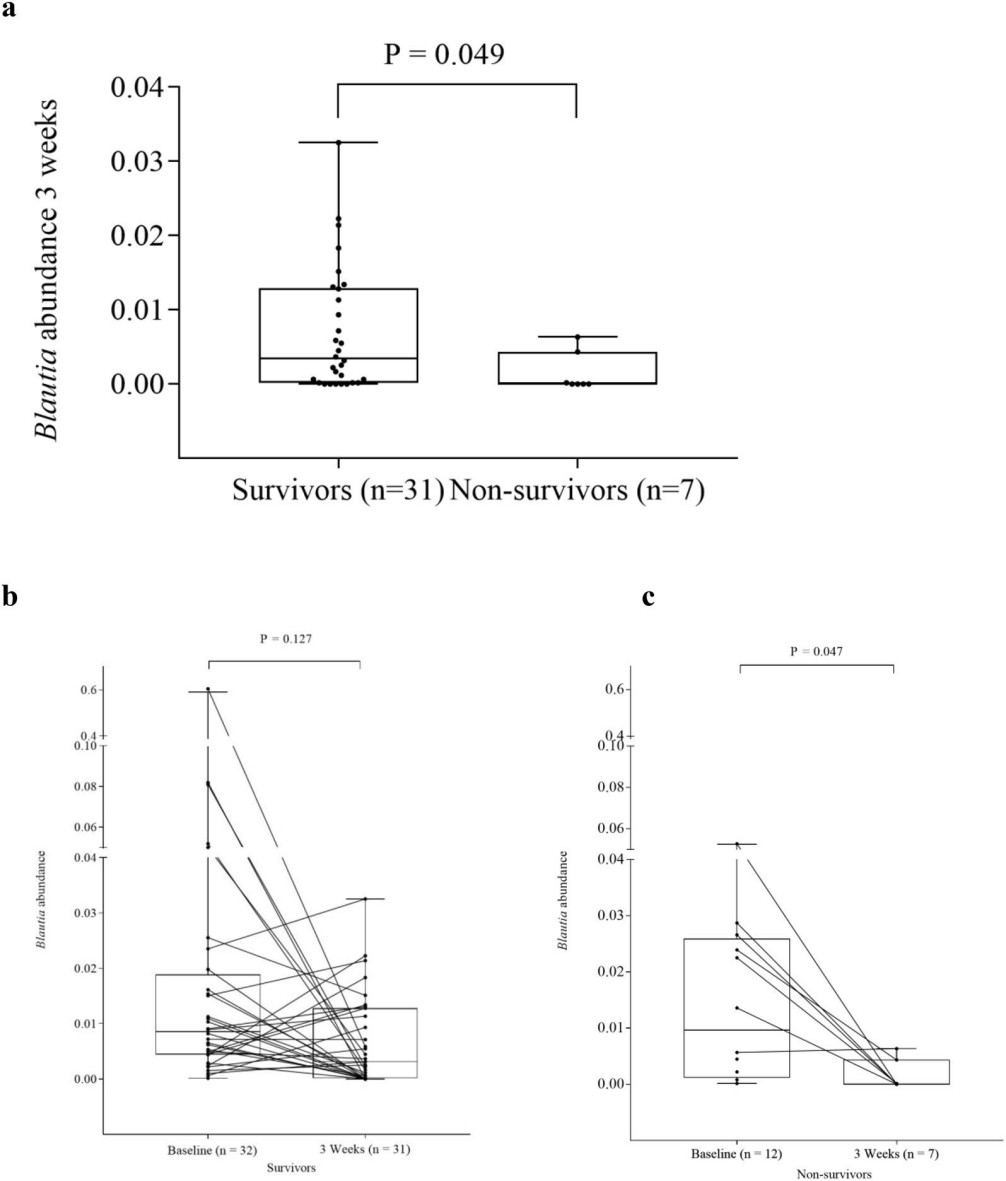
*Blautia* abundance and one-year survival. (**a**) *Blautia* abundance at 3 weeks and one-year survival, (**b**) and (**c**) Drop in *Blautia* abundance from baseline to 3 weeks and one-year survival. Data are *Blautia* abundance given as individual values (dots) and box plots (median and interquartile range) with minimum and maximum values. Lines connect individual data points.

We pooled the intervention and the control group in the analyses of impact of microbiota, SCFAs and biomarkers of gut barrier functions on clinical outcomes (Supplementary Tables S11–S13). The alpha diversity at 3 weeks, but not at baseline, was significantly associated with survival at one year (Fig. 4a, b), with an optimal cut-off of overall survival of observed OTUs > 53 (Supplemental Table S1). When looking at survival curves, observed OTUs > 53 at 3 weeks was associated with both increased overall survival and reduced NRM (*p* = 0.001 and 0.047, respectively), while a similar analysis using the optimal cut-off for Shannon diversity index (> 2.33, Supplementary Table S1) were not significant (*p* = 0.067 and *p* = 0.148, for overall survival and NRM, respectively), Supplementary Fig. S2a–d. The relative loss of observed OTUs from baseline to 3 weeks was larger in non-survivors (median 84%) than in survivors (median 63%), (*p* = 0.035, Fig. 4c). At baseline, the relative abundance of the *Subdoligranulom* genus was lower in patients dying during the first year than in the survivors (*p* = 0.023). At 3 weeks the *Blautia* genus was less abundant in patients who died during follow-up than in those surviving (*p* = 0.049, Fig. 5a). A numerical reduction of *Blautia* abundance in those alive at one year was not significant (*p* = 0.127, Fig. 5b), while a reduction of *Blautia* abundance from baseline to 3 weeks was observed in those who died during follow-up (*p* = 0.047, Fig. 5c). The relative abundance of *Enterococcus* was numerically higher in non-survivors compared with survivors at 3 weeks (Supplementary, Fig. S3). We found no significant associations between aGVHD and alpha diversity or relative abundance of the *Blautia* genus at baseline or at 3 weeks (0.132 ≤ *p* ≤ 0.563 and 0.234 ≤ *P* ≤ 0.657, Supplementary Fig. S4a–c, S5a–e). Q_FDR_ was > 0.75 for all analyses of bacterial taxa.

### Impact of SCFAs on clinical outcomes

Propionic acid at baseline was associated with both death and NRM (*p* = 0.008 and *p* = 0.030, respectively, Supplementary Fig. S6b, S7b). Also, valeric acid and total SCFAs were significantly associated with NRM (*p* = 0.042 and 0.050, respectively, Supplementary Fig. S7f, g). Using the optimal cut-off > 3.05 at baseline (Supplementary Table S1), high propionic acid was associated with improved overall survival (*p* = 0.002) whereas propionic acid > 1.05 at baseline was associated with decreased NRM (*p* < 0.001, Supplementary Fig. S8a, b). Also, significant associations were found for valeric acid and total SCFAs at baseline and NRM (*p* = 0.042 and *p* = 0.052, respectively, Supplementary Fig. S7f, g). Similarly, valeric acid > 0 at baseline and NRM and total SCFAs > 54.14 at baseline, were associated with reduced NRM (*p* = 0.012 and 0.032, respectively, Supplementary Fig. S10a, b). Acetic acid at baseline was the single SCFAs significantly associated with aGVHD (0.038 ≤ *p* ≤ 0.884, Supplementary Fig. S9a–g).

### Impact of markers on gut barrier functions and clinical outcomes

There were no significant associations between I-FABP, LBP and sCD14 at baseline and death (0.172 ≤ *p* ≤ 0.681) and NRM (0.239 ≤ *p* ≤ 0.503, Supplementary Fig. S11, S12). Moreover, no significant associations were observed at 3 weeks between I-FABP, LBP or sCD14 and death (0.177 ≤ *p* ≤ 0.526). At 3 weeks, sCD14 was associated with NRM in contrast to the other gut leakage markers (0.052 ≤ *p* ≤ 0.947, Supplementary, Fig. S14). No significant associations were observed for markers of gut barrier functions at baseline and 3 weeks and aGVHD (0.624 ≤ *p* ≤ 0.853 and 0.385 ≤ *p* ≤ 0.766 respectively, Supplementary Fig. S15, S16).

### Microbial diversity, SCFAs, gut leakage markers and treatment, and disease related factors

With regard to treatment-related factors, we observed a negative correlation between the number of days with i.v. nutrition and alpha diversity measures at 3 weeks (r = − 0.35, *p* = 0.031 for Shannon diversity index and r = – 0.31, *p* = 0.054 for observed OTUs). There was no significant association between i.v. nutrition and *Blautia* abundance at 3 weeks (*p* = 0.254). All patients received i.v. antibiotics in the early post-transplant period, but there were no correlations between number of days with i.v. antibiotics and *Blautia* abundance (r = − 0.23, *p* = 0.160) or measures of alpha diversity (r = − 0.21, *p* = 0.200 for Shannon diversity index and r = − 0.13, *p* = 0.440 for OTUs) at 3 weeks. With regard to impact of disease related factors pre-allo-HSCT for microbial diversity, SCFAs, and markers of gut barrier functions, we observed no association between microbial diversity at baseline and HCT-CI score (0.323 ≤ *p* ≤ 0.485). At 3 weeks, Shannon diversity index was lower in those with intermediate/high, compared too low HCT-CI score (*p* = 0.032). No significant associations were observed for the other measures of microbial diversity and HCT-CI score at 3 weeks (≤ 0.085 *p* ≤ 0.233, Supplementary Fig. S17, S18). There were no significant associations between SCFAs at baseline and HCT-CI score (0.081 ≤ *p* ≤ 0.850, Supplementary Fig. S19). Furthermore, no significant associations were observed for markers of gut barrier function at baseline and 3 weeks, and HCTI-CI score (0.067 ≤ *p* ≤ 0.780 and 0.193 ≤ *p* ≤ 0.841 respectively, Supplementary Fig. S20, S21).

## Discussion

In the present study we observed no impact of the nutritional intervention on gut microbiota, SCFAs, or markers of gut barrier functions. Low diversity and low abundance of *Blautia* genus at 3 weeks and high relative loss of diversity at 3 weeks for the pooled study groups were associated with death the first year, but not with aGVHD. Fecal propionic acid was the single SCFA associated with both death and NRM. Markers of gut barrier functions were less strongly associated with clinical outcomes.

Whether nutritional intervention can modify microbial diversity, improve survival, and reduce the risk of aGVHD in recipients of allo-HSCT is controversial. In our RCT, the intervention had no significant effect on aGVHD or one-year survival^18,19^. In the present study we found that the intervention had no impact on gut microbial diversity, SCFA concentrations, or markers of gut barrier functions, except for a weak, inverse correlation between number of days with PN and alpha diversity. In contrast, one study reported prompt post-HSCT recovery of the gut microbiota and SCFAs in children receiving tube feeding compared to children with PN^17^. Furthermore, tube feeding and several days with oral intake have been associated with reduced aGVHD in two observational studies, both in comparison to PN^28,29^, but energy and protein intakes were not reported in these studies. Microbiota produce SCFAs mainly by fermentation of fiber, but the intervention group in the present study received a fiber-free enteral formula as used in previously studies^30,31^. Similarly, a recent study used a fiber-free formula to optimize EN tolerance and found no difference in microbial diversity between patients receiving predominantly EN versus PN^32^. In contrast, a retrospective case–control analysis suggested that fiber supplementation in allo-HSCT was associated with fewer days of diarrhea and reduced early mortality, but the study design does not allow firm conclusions^33^. Practice patterns for nutritional support during the peritransplant phase varies^15,16^, and observational studies comparing route of feeding may be biased by disease and treatment factors, i.e. the patients with less toxicity and GVHD are those who best tolerate enteral feeding. Overall, there is limited evidence to support nutritional measures focusing on EN as a treatment to modify the gut microbiota to reduce mortality and aGVHD.

A reduction in gut microbial diversity was observed from baseline to 3 weeks in line with several other studies^7,34,35^. Lower microbial diversity and less abundant *Blautia* at 3 weeks were observed in those who died during one year follow-up compared to the survivors. This is also in line with studies showing that microbial diversity around the time of engraftment is associated with mortality following allo-HSCT^7,34^, and that *Blautia* abundance after allo-HSCT is associated with increased lethal GVHD and reduced overall survival^7^. Consistent with available literature we found a numerically increased abundance of *Enterococcus* in non-survivors compared with survivors^8^. In the present study, only data at 3 weeks was associated with clinical outcomes, while the baseline values were not. In contrast, two recent studies analyzed microbiota at several time points and found an association between microbiota disruption and poor clinical outcomes, both before conditioning and after allo-HSCT^5,36^. We observed an association between Shannon diversity index at 3 weeks and HCT-CI. Studies of microbial diversity and diseases-related factors within a larger sample size are therefore needed to determine if microbial diversity is an independent factor on survival.

We found a large reduction in the concentrations of SCFAs from baseline to 3 weeks, possibly caused by both reduced bacterial load and fiber intake. Low concentration of propionic acid at baseline was associated with both death and NRM during follow-up. We further found a correlation between microbial diversity, *Blautia* genus, and butyric acid at 3 weeks. Interestingly, low levels of butyrate and propionate were found in children with aGVHD^11^ and butyric acid may mitigate GVHD in mice^12^.

The gut barrier is compromised after allo-HSCT^37^. In line with one study^3^, we found LBP to be increased from baseline to 3 weeks, which we interpret as a measure of increased bacterial translocation and circulating lipopolysaccharide. The monocyte activation marker sCD14 remained stable, which argues against a systemic inflammatory response driven by microbial products at week 3. We observed a decrease in I-FABP, which has been reported as a biomarker of enterocyte turnover, but with no predictive value, in line with data from other studies^2,4^. In contrast to our observations, higher LBP levels at day + 7 and + 14 post-HSCT have indicated higher probability of aGVHD^3^. Inverse correlation was found for Shannon diversity index and LBP at 3 weeks, but not for sCD14.

Our study had some limitations. Firstly, the subset of patients with fecal samples available may be too limited to detect an impact of the nutritional intervention on microbiota and SCFAs. Secondly, we did not design the intervention to specifically compare route of nutritional support. Our experience is that the sickest patients do not tolerate tube feeding and therefore need parenteral nutrition. Notably, studies comparing route of nutritional support may be biased by disease and treatment factors i.e. the patients with less toxicity and GVHD are those who best tolerate enteral feeding. We therefore focused on the impact of nutritional support per se, compared to other factors known to have impact on microbiota and gut functioning. Additionally, we considered the subset with fecal samples too limited to split for analysis of route of nutritional support. Thirdly, we do not know if oral intake differed between the intervention and control group. We have used an explorative approach also reporting results not withstanding correction for multiple comparisons. Moreover, we focused the discussion of the results primarily on pre-existing hypotheses. Importantly, the relatively limited, but well-characterized subset of patients with fecal samples available did not differ from the total cohort of patients in the original RCT in important characteristics. All patients had a malignant blood disease and received myeloablative conditioning, in contrast to other studies including patients treated with myeloablative conditioning, reduced-intensity conditioning and non-myeloablative conditioning, known to have different impact on microbiota^5,7^.

In conclusion, the nutritional intervention given had no effect on the microbiota, SCFAs, and markers of gut barrier functions, suggesting that the means of intervention must be modified to prevent any negative effects of an altered gut microbiota after allo-HSCT. We found that low bacterial diversity at 3 weeks and relative loss of diversity until 3 weeks as well as low abundance of *Blautia,* independent of pre-HSCT levels, was associated with death within the first year, but with no impact of aGVHD. Propionic acid was the single SCFA associated with both death and NRM. The other SCFAs and circulating markers of gut barrier functions had less impact on clinical outcomes.

## Supplementary Information

Supplementary Information.
