# Supplementary material for: Mortality and microbial diversity after allogeneic hematopoietic stem cell transplantation: secondary analysis of a randomized nutritional intervention trial
Source: Sci Rep. 2021 Jun 2;11:11593. doi: 10.1038/s41598-021-90976-z (PMC8172574; doi:10.1038/s41598-021-90976-z)
Supplement: Supplementary file 1 — Supplementary Information. [file 41598_2021_90976_MOESM1_ESM.pdf]

Supplementary Information for:

Mortality and microbial diversity after allogeneic hematopoietic stem cell transplantation: secondary analysis of a randomized nutritional intervention trial

K. J. Skaarud, J. R. Hov, S. H. Hansen, M. Kummen, J. Valeur, I. Seljeflot, A. Bye, V. Paulsen, K. E. A. Lundin, M. Trøseid, G. E. Tjønnfjord, P. O. Iversen

Methods

When our randomized controlled trial was designed and initially implemented in 2010, the role of gut microbiota in allo-HSCT was scarcely reported, thus we did not collect fecal samples from the initially enrolled patients. We used a standard Fecal Container (Thomas Scientific, Swedesboro, NJ) to store the fecal samples. Reads containing Illumina Universal Adapters or PhiX were discarded using bbduk version 37.55 (BBTools, https://jgi.doe.gov/data-and-tools/bbtools), and the remaining reads were demultiplexed using je version 1.2[1]. Indices, heterogeneity spacers and primers were trimmed with cutadapt version 1.14[2] and the paired end reads were subsequently quality trimmed and merged using bbmerge version 37.55[3]. The merged contigs were quality filtered using default values in Quantitative Insights Into Microbial Ecology (QIIME) version 1.9.1[4] using SortMeRNA version 2.0 [5] through QIIME. OTUs with a number of sequences <0.005% of the total number of mapped sequences were discarded as recommended[6].

Table S1. Cut-off values for microbial diversity, short chain fatty acids, markers of gut barrier functions and clinical outcomes analyzed by comparing Receiver Operating Characteristic curves.

Overall survival

	Baseline			3 weeks		
	AUC (95%CI)	P	Cut-off	AUC (95%CI)	P	Cut-off
Observed OTUs	0.60 (0.44-0.75)	0.28	<=202	0.83 (0.67-0.93)	<0.0001	<=53
Shannon	0.51 (0.35-0.66)	0.92	>4.98	0.77 (0.61-0.89)	0.01	<=2.33
Acetic acid	0.67 (0.50-0.81)	0.11	<=40.17			
Propionic acid	0.80 (0.64-0.91)	0.0001	<=3.05			
Iso-butyric acid	0.61 (0.44-0.76)	0.26	<=1.22			
Butyric acid	0.55 (0.39-0.71)	0.69	<=1.15			
Iso-valeric acid	0.59 (0.42-0.75)	0.38	<=1.71			
Valeric acid	0.59 (0.42-0.75)	0.43	<=0			
Total SCFA	0.69 (0.52-0.82)	0.08	<=58.25			
I-FABP	0.54 (0.44-0.63)	0.52	>1008	0.54 (0.44-0.64)	0.56	<=129
LBP	0.58 (0.48-0.67)	0.16	>12290	0.59 (0.49-0.68)	0.18	>24440
sCD14	0.52 (0.43-0.62)	0.69	<=1618	0.54 (0.44-0.64)	0.55	<=1224

AUC The Area Under the Curve.

Non-relapse mortality

	Baseline			3 weeks		
	AUC (95%CI)	P	Cut-off	AUC (95%CI)	P	Cut-off
Observed OTUs	0.56 (0.40-0.71)	0.64	<132	0.75 (0.58-0.87)	0.0076	<=53
Shannon	0.63 (0.47-0.77)	0.42	>5.45	0.84 (0.68-0.94)	<0.0001	<=2.334
Acetic acid	0.74 (0.57-0.96)	0.075	<=13.05			
Propionic acid	0.84 (0.68-0.94)	0.0021	<=1.05			
Iso-butyric acid	0.74 (0.57-0.86)	0.068	<=0.17			
Butyric acid	0.71 (0.55-0.85)	0.15	<=0.13			
Iso-valeric acid	0.73 (0.56-0.86)	0.089	<=0.42			
Valeric acid	0.81 (0.65-0.92)	0.001	<=0			
Total SCFA	0.80 (0.64-0.91)	0.0065	<=54.14			
I-FABP	0.57 (0.47-0.66)	0.39	>717	0.51 (0.41-0.60)	0.95	<=129
LBP	0.58 (0.49-0.68)	0.21	>13301	0.56 (0.46-0.66)	0.55	>24525
sCD14	0.55 (0.45-0.64)	0.53	>1894	0.67 (0.57-0.75)	0.060	<=1830

AUC The Area Under the Curve.

Table S2. Clinical characteristics at inclusion of the total study cohort (n = 117)

	Intervention (n = 57)	Control (n = 60)
Median (range) age (years)	45 (19-65)	41 (18-62)
Female	20 (35)	25 (42)
Diagnosis		
AML	36 (63)	31 (51)
ALL	6 (10)	10 (17)
CML	2 (4)	7 (12)
CMML	3 (5)	3 (5)
aCML	1 (2)	-
MDS	6 (11)	5 (8)
APL	1 (2)	-
ABL	-	1 (2)
AUL	-	1 (2)
MCL	-	1 (2)
MF	2 (4)	-
MS	-	1 (2)
Donor		
HLA - identical sibling	17 (30)	13 (22)
HLA - identical unrelated	40 (70)	47 (78)
Stem-cell source		
Bone marrow	25 (44)	27 (45)
Peripheral - blood hematopoietic cells	32 (56)	33 (55)
Sex mismatch*	17 (30)	10 (17)
Conditioning		
Busulphan + Cyclophosphamide	56 (98)	56 (93)
TBI + Cyclophosphamide	1 (2)	4 (7)
HCTI - CI risk groups		
Low risk	42 (74)	45 (75)
Intermediate risk	8 (14)	10 (17)
High risk	7 (12)	5 (8)

Table S2. Clinical characteristics at inclusion of the total study cohort (n = 117)

	Intervention (n = 57)	Control (n = 60)
EBMT score		
0-3	33 (58)	36 (60)
4	14 (24)	14 (23)
5-7	10 (18)	10 (17)
Performance status ECOG		
0	55 (96)	54 (90)
1	2 (4)	6 (10)
BMI		
Underweight	2 (4)	4 (7)
Normal weight	31 (54)	27 (45)
Overweight	17 (30)	26 (43)
Moderately obese	4 (7)	3 (5)
Severely obese	3 (5)	0 (0)

Values are numbers (%) unless otherwise stated. AML Acute myeloid leukemia, ALL Acute lymphocytic leukemia, CML Chronic myeloid leukemia, CMML Chronic myelomonocytic leukemia, aCML atypical Chronic myeloid leukemia, MDS Myelodysplastic syndrome, APL Acute promyelocytic leukemia, ABL Acute basophilic leukemia, AUL Acute undifferentiated leukemia, MCL Mast cell leukemia, MF Myelofibrosis, MS Myeloid sarcoma, TBI Total body irradiation, HCTI-CI Hematopoietic Cell Transplantation-specific comorbidity index, EBMT score European Group for Blood and Marrow Transplantation score, ECOG Eastern Cooperative Oncology Group, [*]Sex mismatch was defined as female donor-to-male recipient.

Results

Figure S1. Kaplan-Meier plot for one-year overall survival. P-value derived from Log-Rank test.

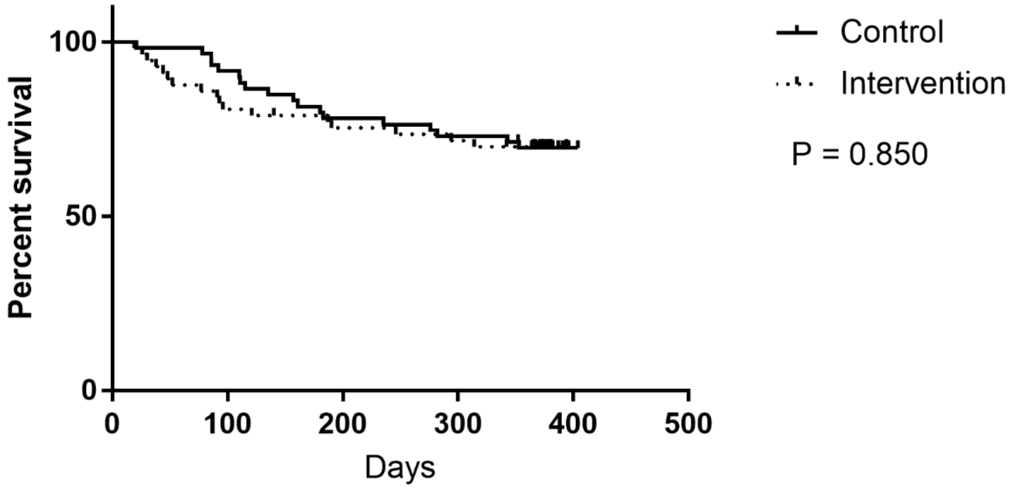

Table S3. Reduction in microbial diversity from baseline to 3 weeks in the two study groups

	Intervention			Control		
	Baseline (n = 22)	3 Weeks (n = 19)	P value	Baseline (n = 22)	3 Weeks (n = 19)	P value
Observed OTUs	192 (104-435)	54 (13-167)	< 0.001	209 (89-371)	68 (25-229)	< 0.001
Shannon index	4.91 (3.26-6.41)	2.74 (0.72-5.11)	< 0.001	4.73 (2.97-6.24)	2.98 (0.91-5.44)	0.001
Blautia	0.01 (0.00-0.60)	0.00 (0.00-0.03)	0.048	0.01 (0.00-0.08)	0.00 (0.00-0.02)	0.113
Enterococcus	0.00 (0.00-0.00)	0.00 (0.00-0.89)	0.001	0.00 (0.00-0.00)	0.00 (0.00-0.06)	0.021

Values are given as median (range), P values derived from Wilcoxon signed rank test.

Table S4. Microbial diversity between the two study groups at baseline and after 3 weeks

	Baseline		3 weeks		
	Intervention (n = 22)	Control (n = 22)	Intervention (n = 19)	Control (n = 19)	P value
Observed OTUs	192 (104-435)	209 (89-371)	54 (13-167)	68 (25-229)	0.293
Shannon index	4.91 (3.26-6.41)	4.73 (2.97-6.24)	2.74 (0.72-5.11)	2.98 (0.91-5.44)	0.737
Blautia	0.01 (0.00-0.60)	0.01 (0.00-0.08)	0.00 (0.00-0.03)	0.00 (0.00-0.02)	0.316
Enterococcus	0.00 (0.00-0.00)	0.00 (0.00-0.00)	0.00 (0.00-0.89)	0-00 (0.00-0.06)	0.097

Values are given as median (range), P value from Mann-Whitney U test.

Table S5. Reduction in fecal short chain fatty acids from baseline to 3 weeks in the two study groups

	Intervention			Control		
	Baseline (n = 18)	3 Weeks (n = 11)	P value	Baseline (n = 21)	3 Weeks (n = 5)	P value
Acetic acid	35.17 (12.82-58.89)	17.65 (5.39-43.18)	0.008	39.88 (6.41-77.2)	16.69 (4.46-54.53)	0.080
Propionic acid	4.13 (0.00-18.37)	2.90 (0.00-8.06)	0.008	4.74 (0.19-17.64)	2.29 (0.00-12.36)	0.500
Iso-butyric acid	1.19 (0.13-2.27)	0.24 (0.00-1.06)	0.033	0.91 (0.00-2.89)	0.39 (0.18-1.42)	0,080
Butyric acid	5.97 (0.13-16.34)	1.20 (0.00-4.12)	0.013	5.65 (0.00-34.44)	0.89 (0.30-7.53)	0.043
Iso-valeric acid	1.55 (0.22-3.66)	0.32 (0.00-1.23)	0.013	1.22 (0.13-3.47)	0.54 (0.21-2.17)	0.138
Valeric acid	0.14 (0.00-3.16)	0.00 (0.00-0.18)	0.018	0.18 (0.00-2.89)	0.00 (0.00-0.12)	0.043
Total short chain fatty acids	51.38 (14.39-101.20)	27.00 (5.39-46.63)	0.006	39.88 (6.41-77.2)	23.21 (6.27-68.04)	0.043

Values are mmol/kg given as median (range), P values derived from Wilcoxon signed rank test.

Table S6. Fecal short chain fatty acids between the two study groups at baseline and at 3 weeks

	Baseline		3 weeks		
	Intervention (n = 18)	Control (n = 21)	Intervention (n = 11)	Control (n = 5)	P value
Acetic acid	35.17 (12.82-58.89)	39.88 (6.41-77.2)	17.65 (5.39-43.18)	16.69 (4.46-54.53)	0.777
Propionic acid	4.13 (0.00-18.37)	4.74 (0.19-17.64)	2.90 (0.00-8.06)	2.29 (0.00-12.36)	0.955
Iso-butyric acid	1.19 (0.13-2.27)	0.91 (0.00-2.89)	0.24 (0.00-1.06)	0.39 (0.18-1.42)	0.234
Butyric acid	5.97 (0.13-16.34)	5.65 (0.00-34.44)	1.20 (0.00-4.12)	0.89 (0.30-7.53)	0.461
Iso-valeric acid	1.55 (0.22-3.66)	1.22 (0.13-3.47)	0.32 (0.00-1.23)	0.54 (0.21-2.17)	0.335
Valeric acid	0.14 (0.00-3.16)	0.18 (0.00-2.89)	0.00 (0.00-0.18)	0.00 (0.00-0.12)	0.491
Total short chain fatty acids	51.38 (14.39-101.20)	63.52 (7.19-135.17)	27.00 (5.39-46.63)	23.21 (6.27-68.04)	0.692

Values are mmol/kg given as median (range), P value from Mann-Whitney U test.

Table S7. Reduction in markers of gut barrier functions from baseline to 3 weeks in the two study groups

	Intervention			Control		
	Baseline (n = 55)	3 Weeks (n = 53)	P value	Baseline (n = 59)	3 Weeks (n = 55)	P value
I-FABP (pg/ml)	397 (122-1442)	242 (47-3820)	0.003	473 (60-2346)	204 (47-1373)	< 0.001
LBP (ng/ml)	13794 (4410-28500)	18618 (4905-39149)	< 0.001	13389 (7098-31896)	18618 (4905-39149)	< 0.001
sCD14 (ng/ml)	1651.9 (881.9-3213.8)	21750.5 (990.4-3010.5)	0.437	1820.6 (987.7-2842.6)	1829.7 (905.9-2853.3)	0.651

Values are given as median (range), P values derived from Wilcoxon signed rank test.

Table S8. Markers of gut barrier functions between the two study groups at baseline and at 3 weeks

	Baseline		3 weeks		
	Intervention (n = 55)	Control (n = 59)	Intervention (n = 53)	Control (n = 55)	P value
I-FABP (pg/ml)	397 (122-1442)	473 (60-2346)	242 (47-3820)	204 (47-1373)	0.503
LBP (ng/ml)	13794 (4410-28500)	13389 (7098-31896)	18618 (4905-39149)	18618 (4905-39149)	0.823
sCD14 (ng/ml)	1651.9 (881.9-3213.8)	1820.6 (987.7-2842.6)	21750.5 (990.4-3010.5)	1829.7 (905.9-2853.3)	0.043

Values are given as median (range), P values derived from Mann-Whitney U test.

Table S9. Correlations among alpha diversity, short chain fatty acids and markers of gut barrier function at baseline

	OTUs		Shannon		*Blautia* abundance	
	r	P value	r	P value	r	P value
Short chain fatty acid*						
Acetic acid	0.14	0.395	0.17	0.308	- 0.05	0.759
Propionic acid	0.22	0.186	- 0.03	0.851	- 0.39	0.016
Iso-butyric acid	0.17	0.136	0.16	0.319	- 0.16	0.348
Butyric acid	0.30	0.076	0.24	1.160	0.02	0.895
Iso-valeric acid	0.24	0.152	0.27	0.101	- 0.26	0.126
Valeric acid	0.41	0.012	0.17	0.305	- 0.28	0.091
Total short chain fatty acid	0.30	0.070	0.21	0.224	- 0.10	0.552
Markers of gut barrier functions						
I-FABP (pg/ml)	0.21	0.167	0.31	0.043	0.03	0.870
LPB (ng/ml)	0.25	0.099	0.13	0.399	- 0.28	0.062
sCD14 (ng/ml)	- 0.06	0.693	- 0.07	0.659	- 0.26	0.090

*Values are (mmol/kg), r and P values are based on Spearman's correlation coefficients.

Table S10. Correlations among alpha diversity, short chain fatty acids and markers of gut barrier functions at 3 weeks

	OTUs		Shannon		*Blautia* abundance	
	r	P value	r	P value	r	P value
Short chain fatty acid*						
Acetic acid	0.49	0.061	0.59	0.020	0.32	0.249
Propionic acid	0.63	0.011	0.61	0.017	0.36	0.185
Iso-butyric acid	- 0.02	0.950	0.34	0.221	0.20	0.467
Butyric acid	0.51	0.050	0.63	0.011	0.57	0.027
Iso-valeric acid	0.18	0.534	0.31	0.259	0.22	0.423
Valeric acid	0.27	0.329	0.32	0.239	0.12	0.683
Total short chain fatty acid	0.61	0.016	0.66	0.007	0.47	0.078
Markers of gut barrier functions						
I-FABP (pg/ml)	0.27	0.098	0.45	0.005	0.39	0.017
LPB (ng/ml)	- 0.24	0.140	- 0.35	0.032	- 0.20	0.239
sCD14 (ng/ml)	- 0.22	0.184	- 0.35	0.034	- 0.07	0.659

*Values are (mmol/kg), r and P values are based on Spearman's correlation coefficients.

Table S11. Microbial diversity at baseline and at 3 weeks for the pooled study cohort

	Baseline (n = 44)	3 weeks (n = 38)	P value
Observed OTUs	198 (89-435)	57 (13-229)	< 0.001
Shannon index	4.84 (2.97-6.41)	2.80 (0.72-5.44)	< 0.001
Blautia	0.01 (0.00-0.60)	0.00 (0.00-0.03)	0.015

Values are given as median (range), P value from Wilcoxon signed rank test.

Table S12. Fecal short chain fatty acids at baseline and at 3 weeks for the pooled study cohort

	Baseline (n = 39)	3 weeks (n = 16)	P value
Acetic acid	37.12 (6.41-77.20)	17.17 (4.46-54.53)	0.002
Propionic acid	4.74 (0.00-18.37)	2.60 (0.00-12.36)	0.008
Iso-butyric acid	0.92 (0.00-2.89)	0.27 (0.00-1.42)	0.008
Butyric acid	5.65 (0.00-34.44)	1.05 (0.00-7.53)	0.001
Iso-valeric acid	1.35 (0.13-3.66)	0.33 (0.00-2.17)	0.004
Valeric acid	0.15 (0.00-3.16)	0.00 (0.00-1.18)	0.002
Total short chain fatty acids	62.45 (28.24-101.20)	25.11 (5.39-68.04)	0.001

Values (mmol/kg) are given as median (range), P value from Wilcoxon signed rank test.

Table S13. Markers of gut barrier functions at baseline and at 3 weeks for the pooled study cohort

	Baseline (n = 114)	3 weeks (n = 108)	P value
I-FABP (pg/ml)	432 (60-2346)	214 (47-3820)	< 0.001
LBP (ng/ml)	13695 (4410-31896)	18805 (4905-43176)	< 0.001
sCD14 (ng/ml)	1745 (882-3214)	1803 (906-3011)	0.444

Values are given as medians (range). P value from Wilcoxon signed rank test.

Figure S2. Kaplan-Meier plots for high and low alpha diversity at 3 weeks and overall survival, and non-relapse mortality (NRM). **a** High- and low observed OTUs and overall survival, **b** High- and low observed OTUs and NRM, **c** High- and low Shannon diversity index and overall survival, **d** High- and low Shannon diversity index and NRM. P-value derived from Log-Rank test.

a

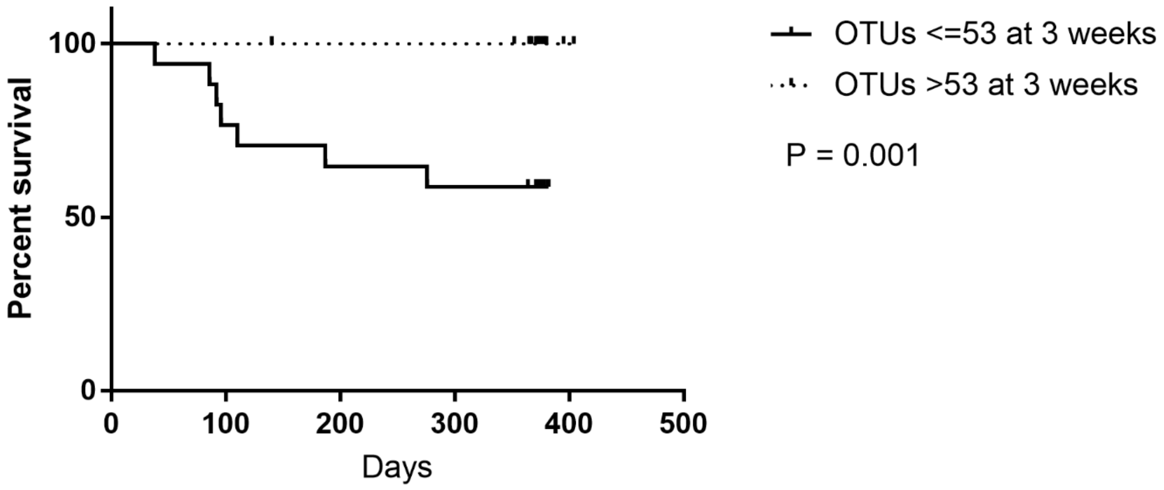

b

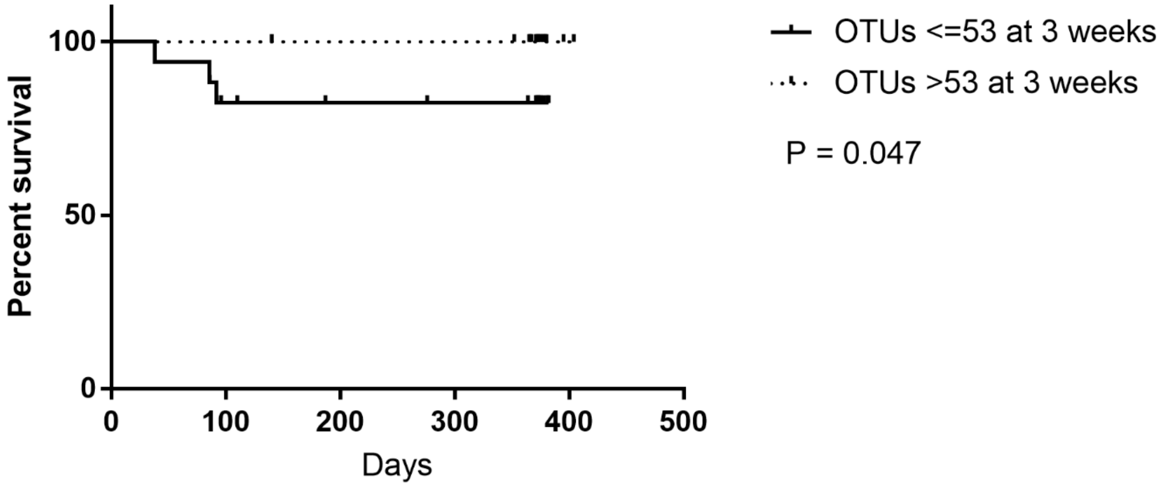

c

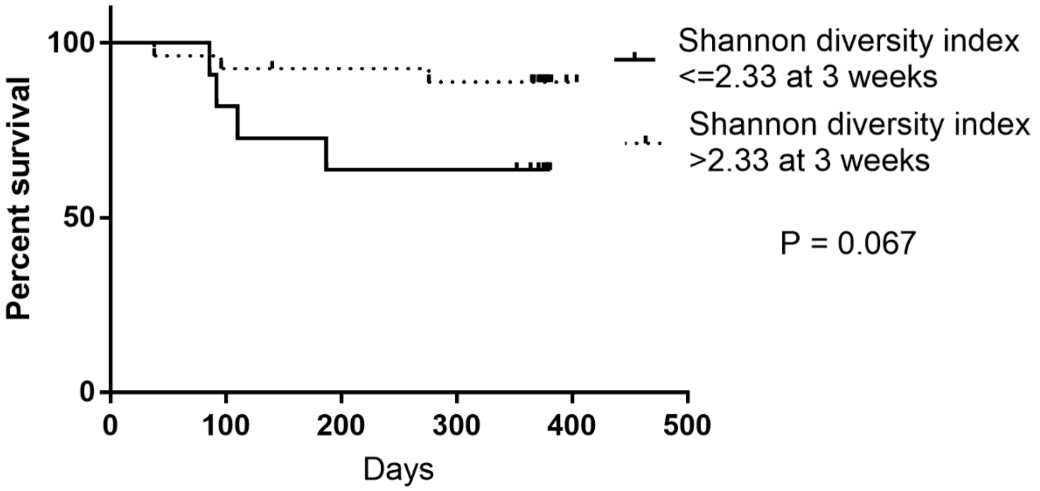
100
50
0
Percent survival
0
100
200
300
400
500
Days
Shannon diversity index <=2.33 at 3 weeks
Shannon diversity index >2.33 at 3 weeks
P = 0.067

d

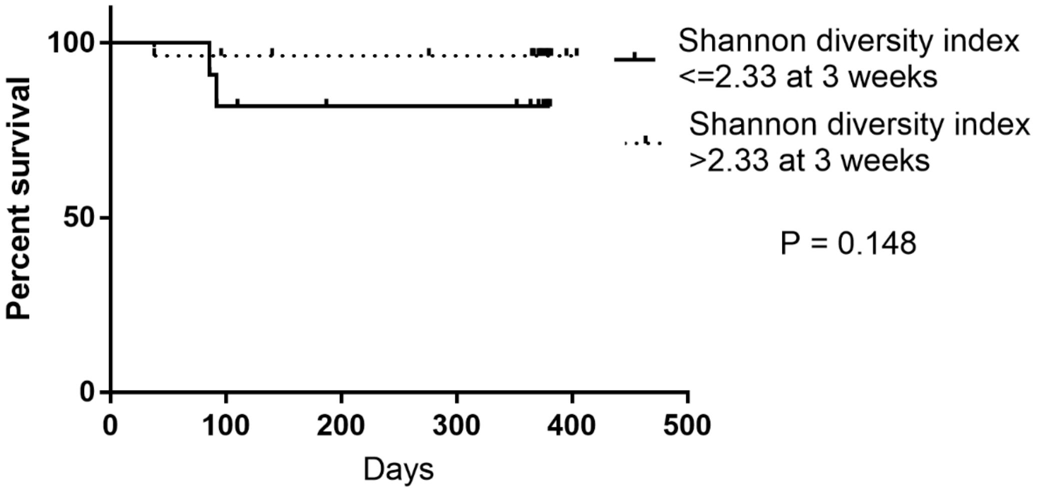
100
50
0
Percent survival
0
100
200
300
400
500
Days
Shannon diversity index <=2.33 at 3 weeks
Shannon diversity index >2.33 at 3 weeks
P = 0.148

Figure S3. *Enterococcus* abundance at 3 weeks and survival. Data are Enterococcus abundance given as box plots (median and interquartile range) with minimum and maximum values. P values derived from Mann-Whitney U test.

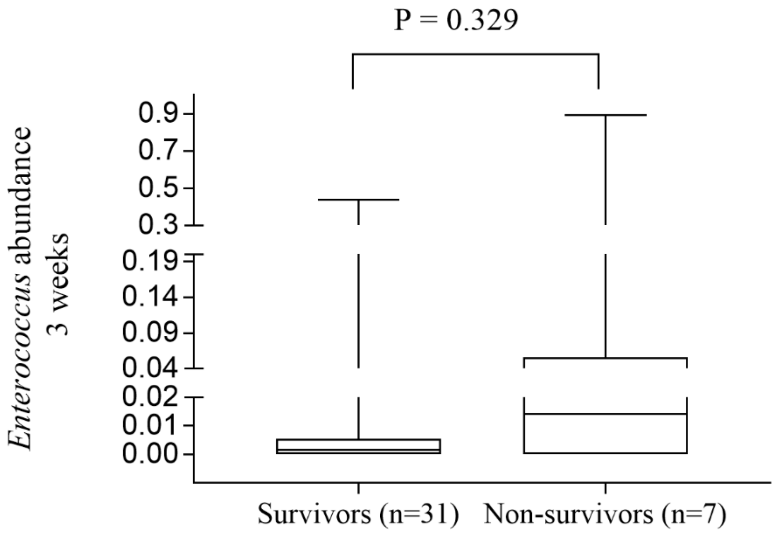

Figure S4. The relationship between microbial diversity at baseline and aGVHD. **a** Observed OTUs and aGVHD, **b** Shannon diversity index and aGVHD, **c** *Blautia* abundance and aGVHD. Data are observed OTUs, Shannon diversity index and *Blautia* abundance given as individual values (dots) and as box plots (median and interquartile range) with minimum and maximum values. P values derived from Mann-Whitney U test.

a

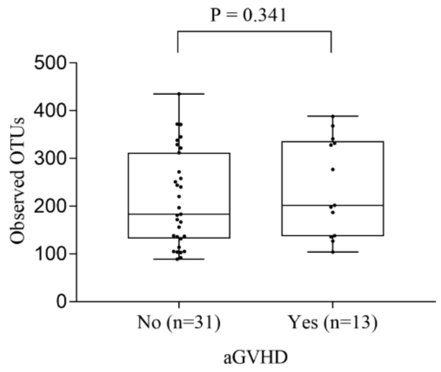

b

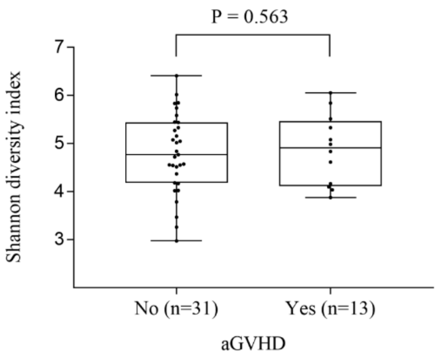

c

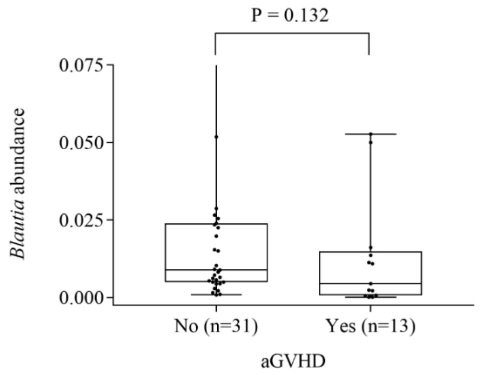

Figure S5. The relationship between microbial diversity at 3 weeks and aGVHD. **a** Observed OTUs and aGVHD, **b** Reduction in observed OTUs and aGVHD, **c** Shannon diversity index and aGVHD, **d** *Blautia* abundance and aGVHD, **e** reduction in *Blautia* abundance and aGVHD. Data are observed OTUs, reduction in observed OTUs, Shannon diversity index, *Blautia* abundance, reduction in *Blautia* abundance given as individual values (dots) and as box plots (median and interquartile range) with minimum and maximum values. P values derived from Mann-Whitney U test.

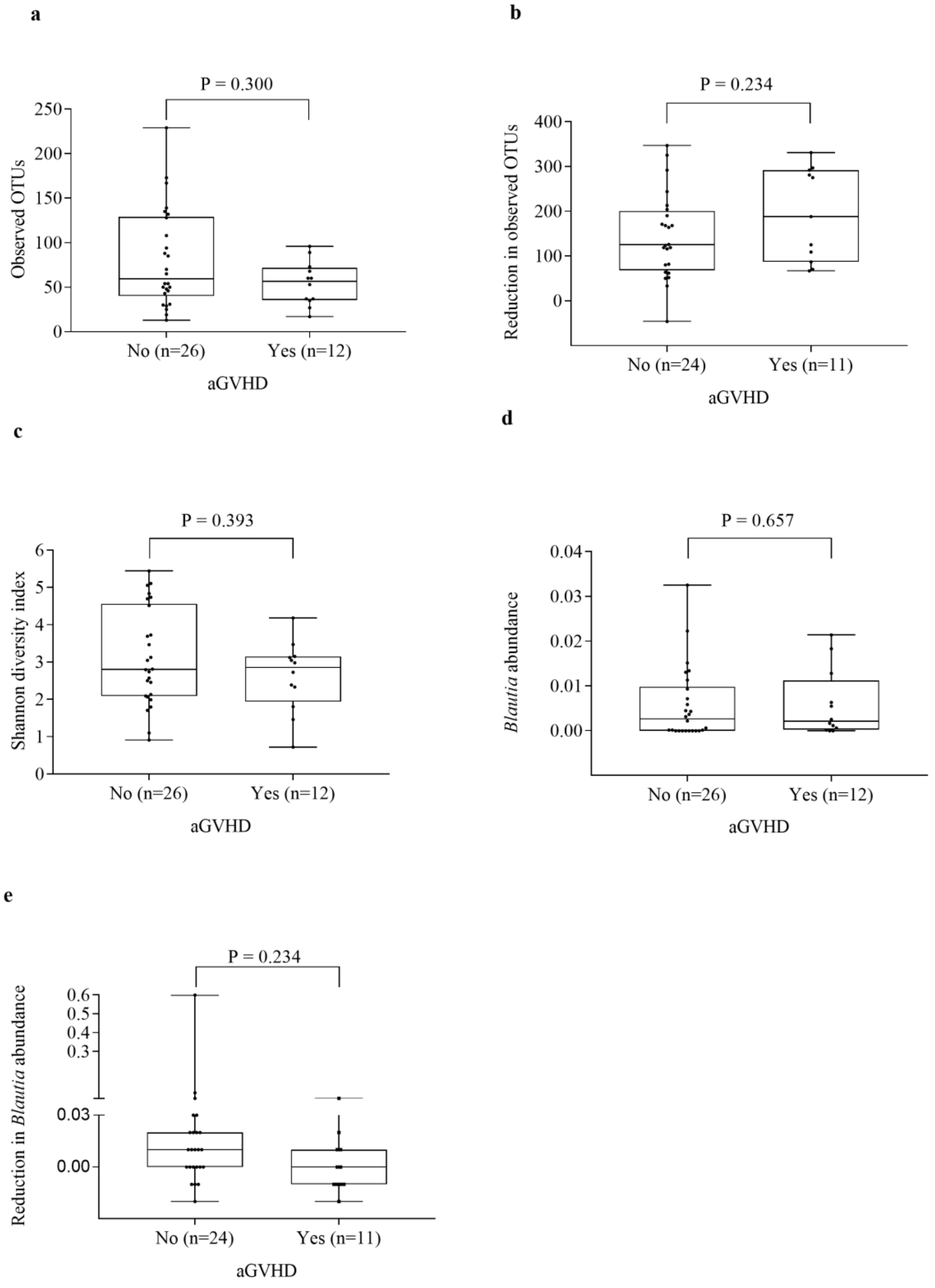

Figure S6. The relationship between SCFAs at baseline and survival. **a** Acetic acid and survival, **b** Propionic acid and survival, **c** Iso-butyric acid and survival, **d** Butyric acid and survival, **e** Iso-valeric acid and survival, **f** Valeric acid and survival, **g** total SCFAs and survival. Data are Acetic acid, Propionic acid, Iso-butyric acid, Butyric acid, Iso-valeric acid, Valeric acid and total SCFAs given as individual values (dots) and as box plots (median and interquartile range) with minimum and maximum values. P values derived from Mann-Whitney U test.

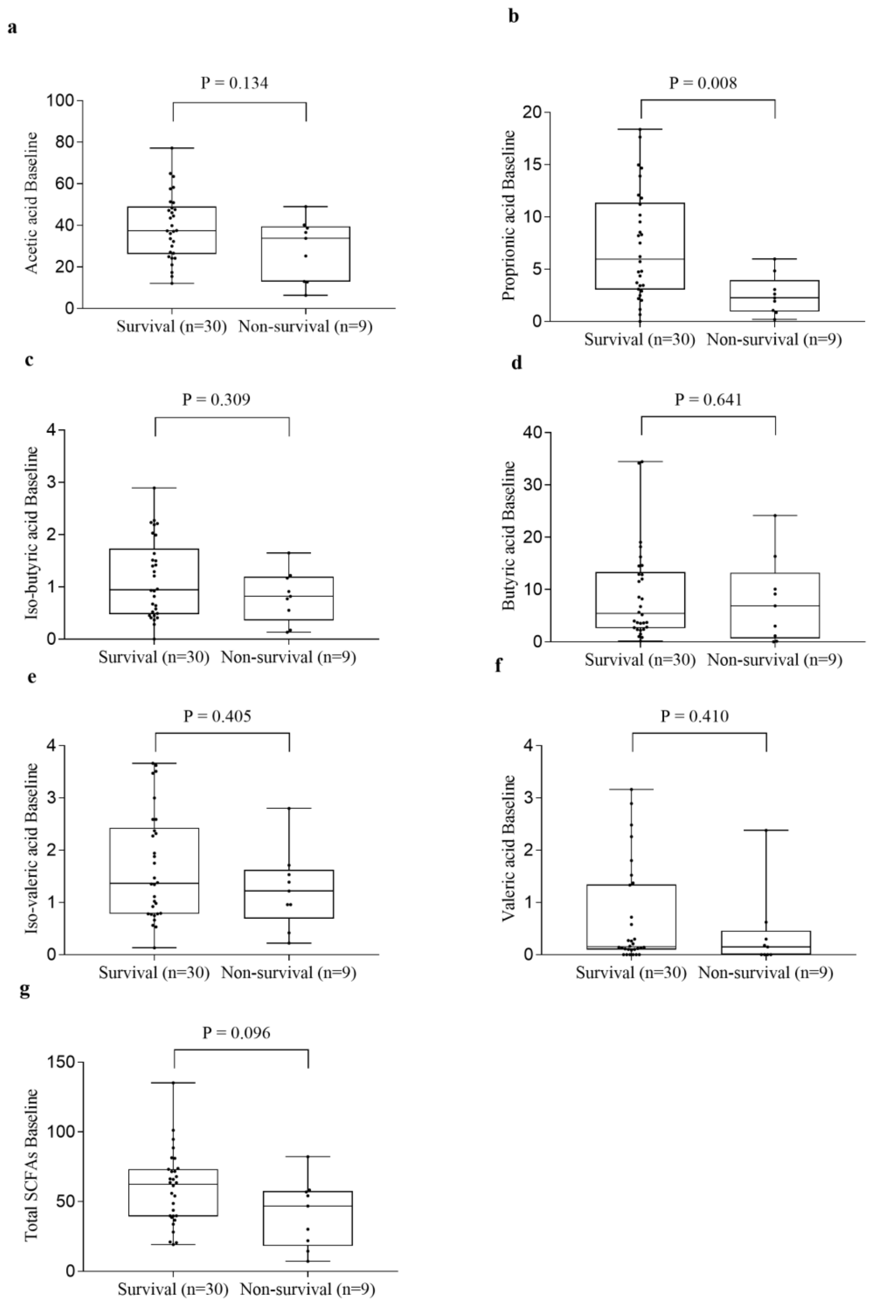

Figure S7. The relationship between SCFAs at baseline and non-relapse mortality (NRM). **a** Acetic acid and NRM, **b** Propionic acid and NRM, **c** Iso-butyric acid and NRM, **d** Butyric acid and NRM, **e** Iso-valeric acid and NRM, **f** Valeric acid and NRM, **g** total SCFAs and NRM. Data are Acetic acid, Propionic acid, Iso-butyric acid, Butyric acid, Iso-valeric acid, Valeric acid and total SCFAs given as individual values (dots) and as box plots (median and interquartile range) with minimum and maximum values. P values derived from Mann-Whitney U test.

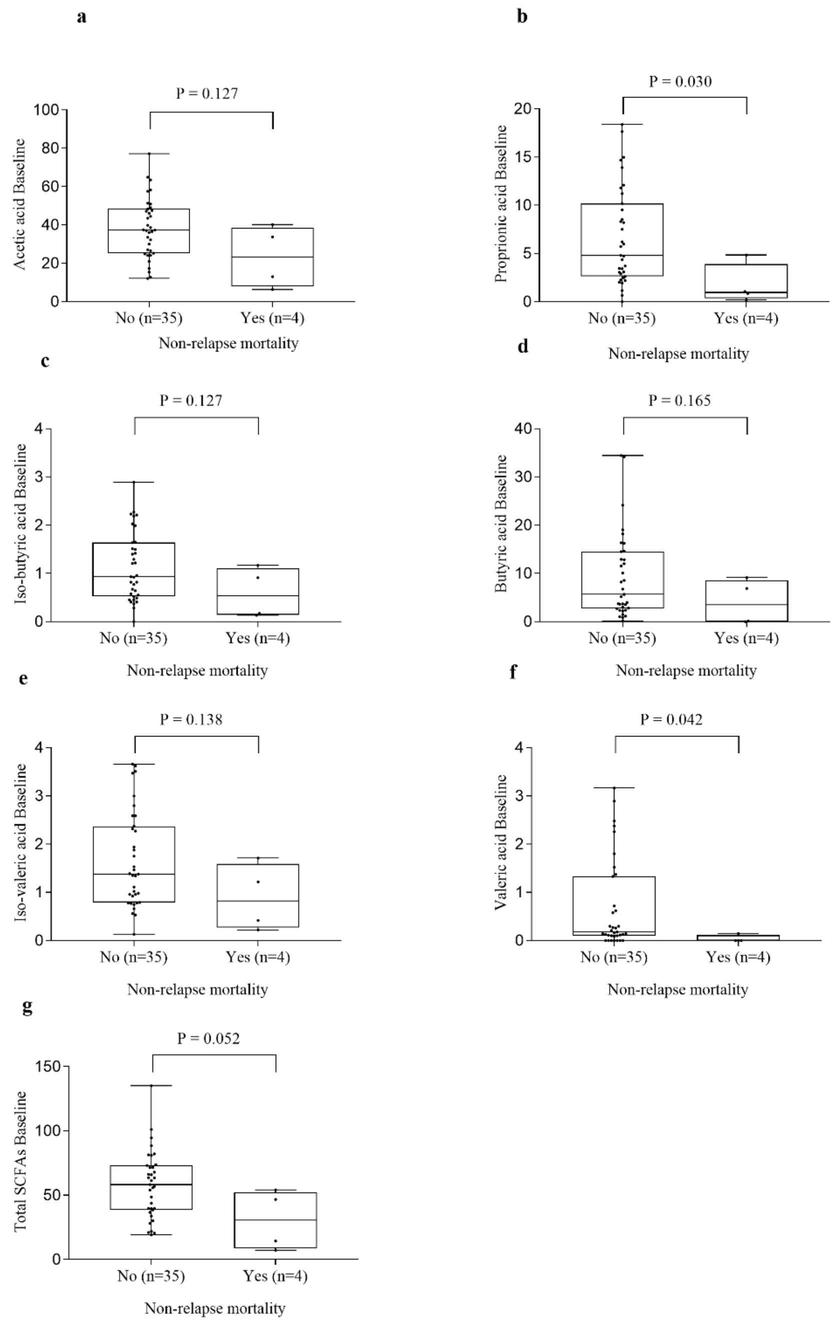

Figure S8 Kaplan-Meier plot for overall survival and non-relapse mortality (NRM) and cut-off values for propionic acid at baseline. **a** Overall survival and propionic acid, **b** NRM and propionic acid at baseline. P-value derived from Log-Rank test.

a

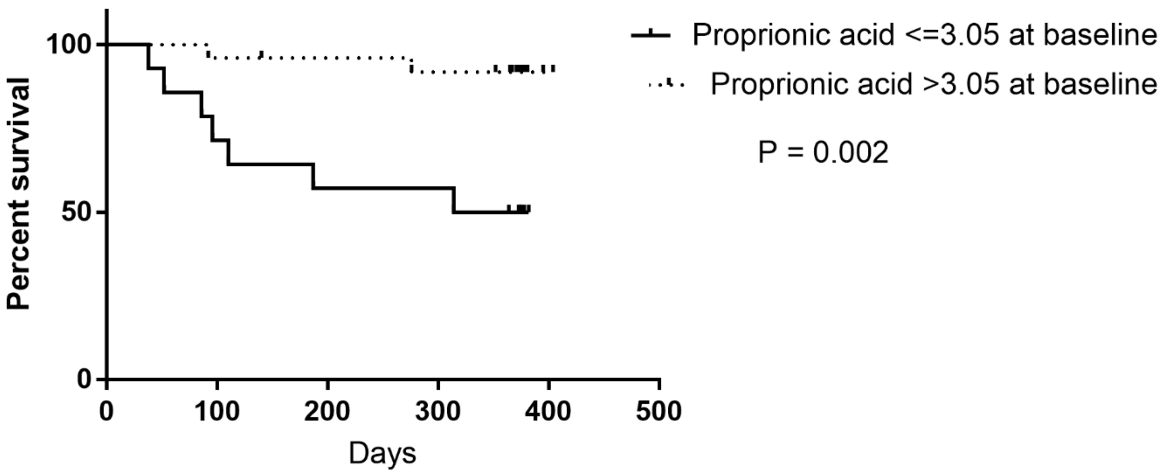

b

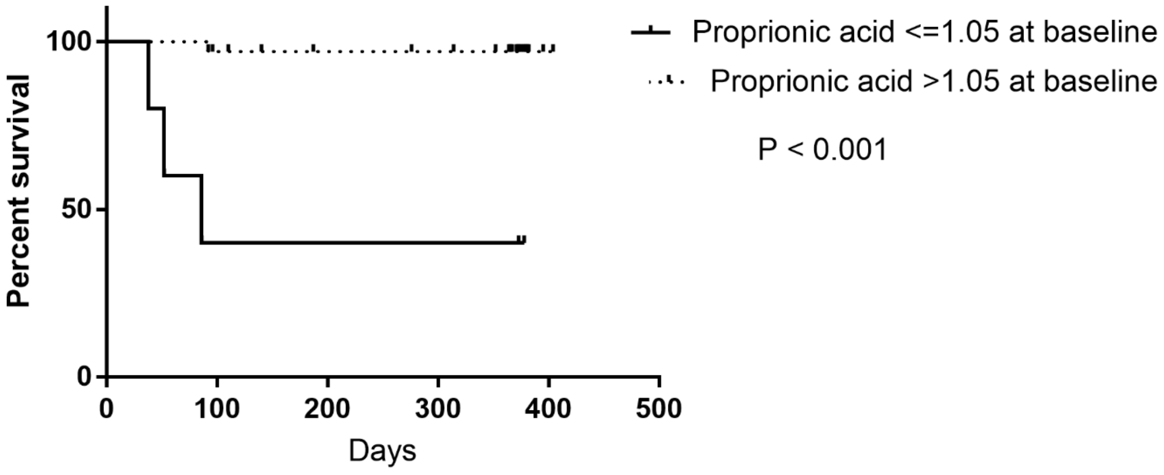

Figure S9. The relationship between SCFAs at baseline and aGVHD. **a** Acetic acid and aGVHD, **b** Propionic acid and aGVHD, **c** Iso-butyric acid and aGVHD, **d** Butyric acid and aGVHD, **e** Iso-valeric acid and aGVHD, **f** Valeric acid and aGVHD, **g** total SCFAs and aGVHD. Data are Acetic acid, Propionic acid, Iso-butyric acid, Butyric acid, Iso-valeric acid, Valeric acid and total SCFAs given as individual values (dots) and as box plots (median and interquartile range) with minimum and maximum values. P values derived from Mann-Whitney U test.

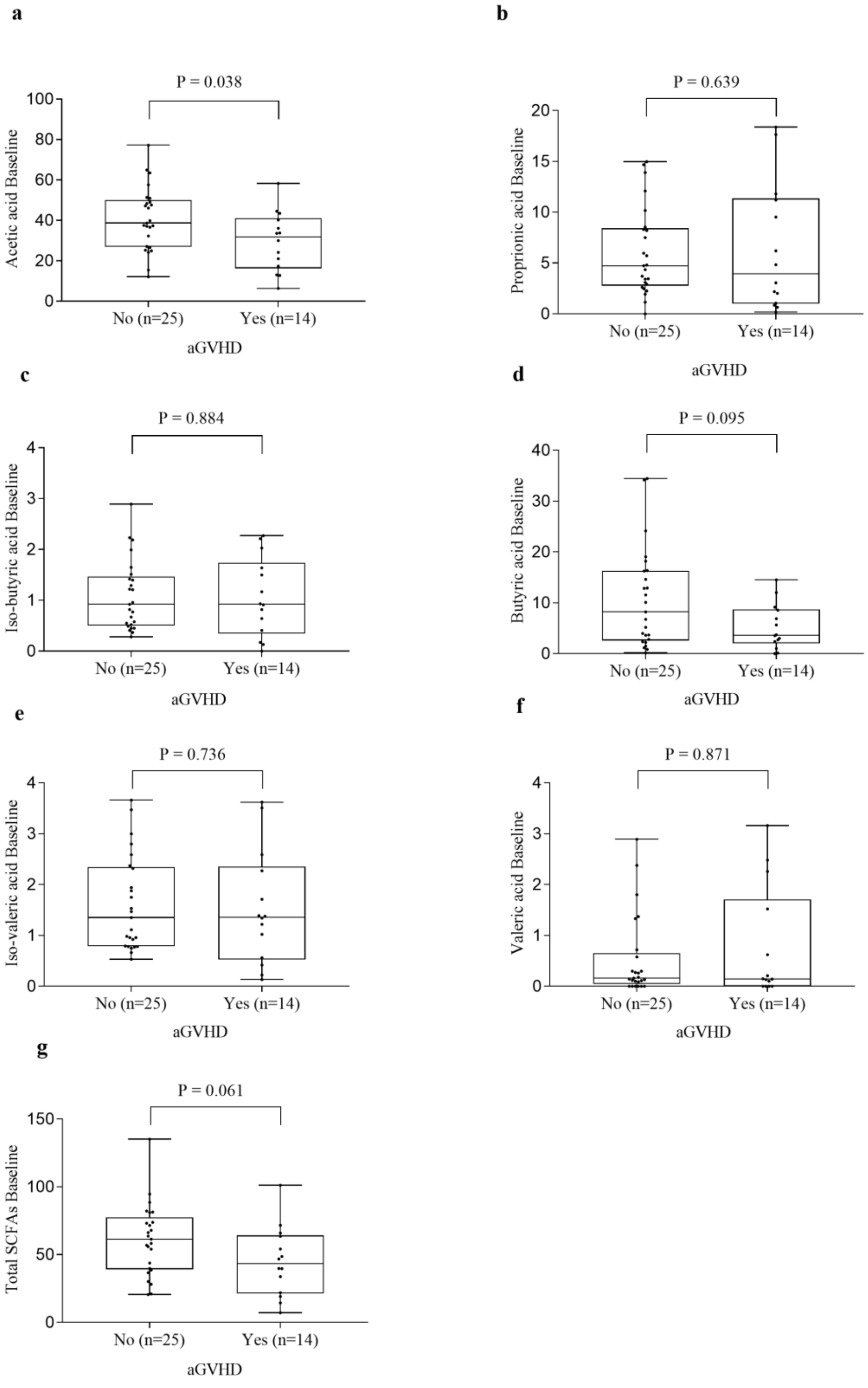

Figure S10 Kaplan-Meier plot for non-relapse mortality (NRM) and valeric acid and total SCFAs at baseline. P-value derived from Log-Rank test.

a

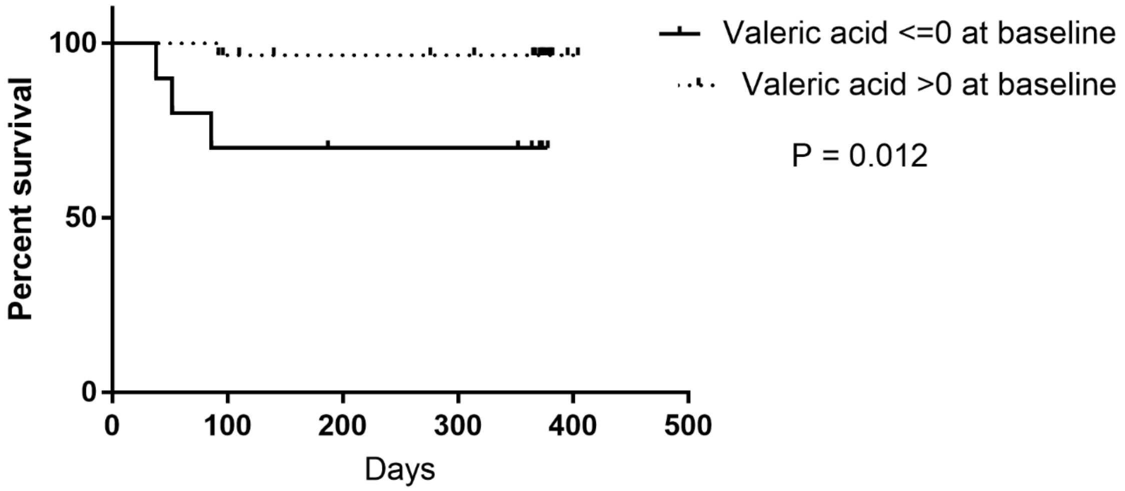

b

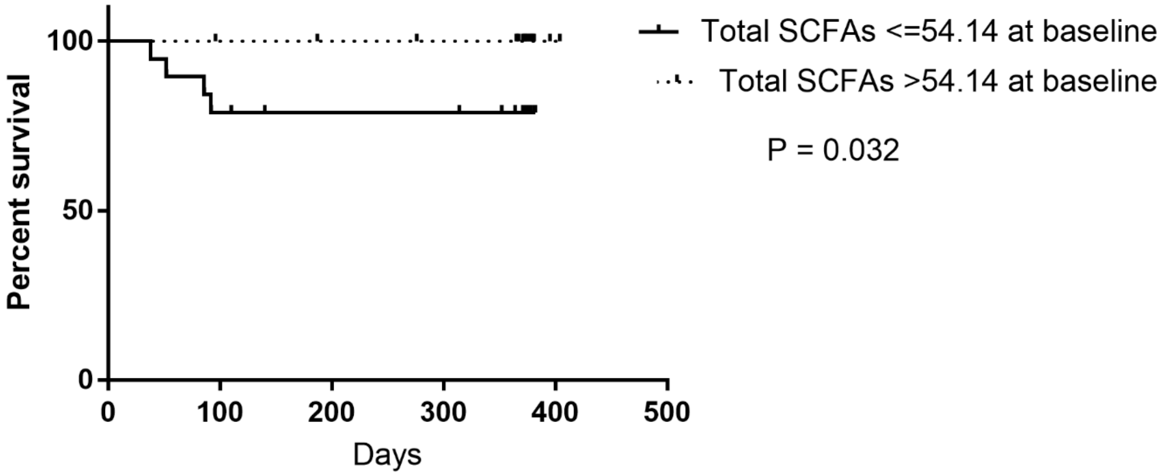

Figure 11. The relationship between markers of gut barrier functions at baseline and survival. **a** I-FABP and survival, **b** LBP and survival, **c** sCD14 and survival. Data are I-FABP, LBP and sCD14 given as individual values (dots) and as box plots (median and interquartile range) with minimum and maximum values. P values derived from Mann-Whitney U test.

a

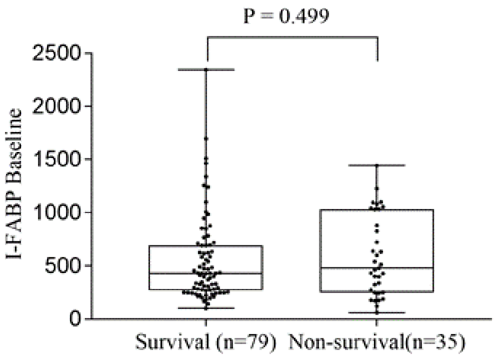

b

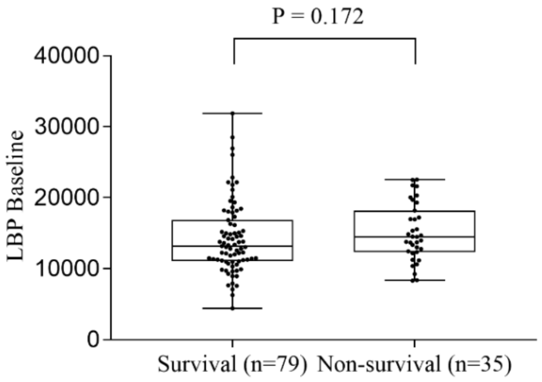

c

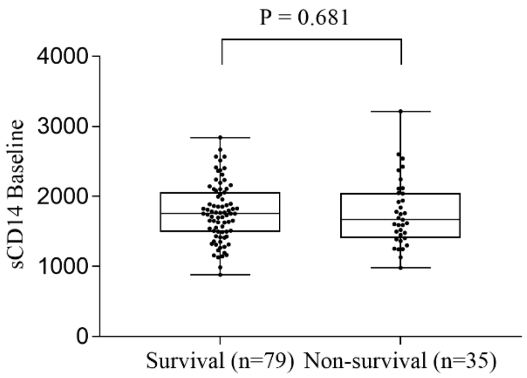

Figure S12. The relationship between gut leakage markers at 3 weeks and survival. **a** I-FABP and survival, **b** LBP and survival, **c** sCD14 and survival. Data are I-FABP, LBP and sCD14 given as individual values (dots) and as box plots (median and interquartile range) with minimum and maximum values. P values derived from Mann-Whitney U test.

a

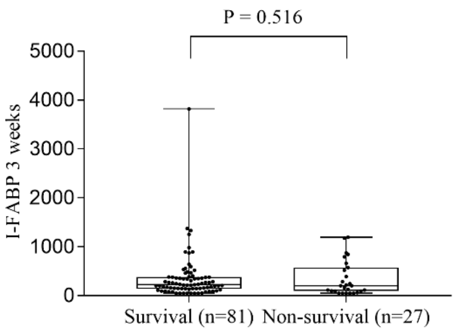

b

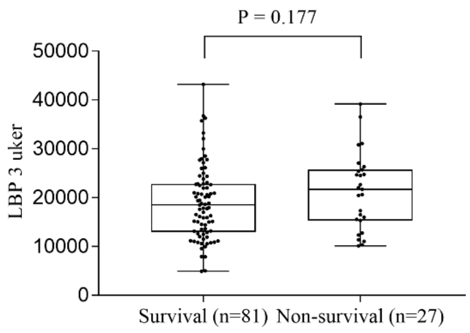

c

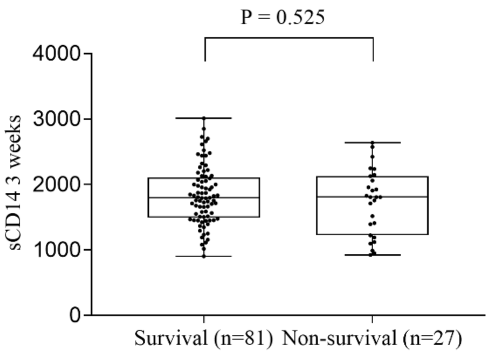

Figure 13. The relationship between markers of gut barrier functions at baseline and non-relapse mortality (NRM). **a** I-FABP and NRM, **b** LBP and NRM, **c** sCD14 and NRM. Data are I-FABP, LBP and sCD14 given as individual values (dots) and as box plots (median and interquartile range) with minimum and maximum values. P values derived from Mann-Whitney U test.

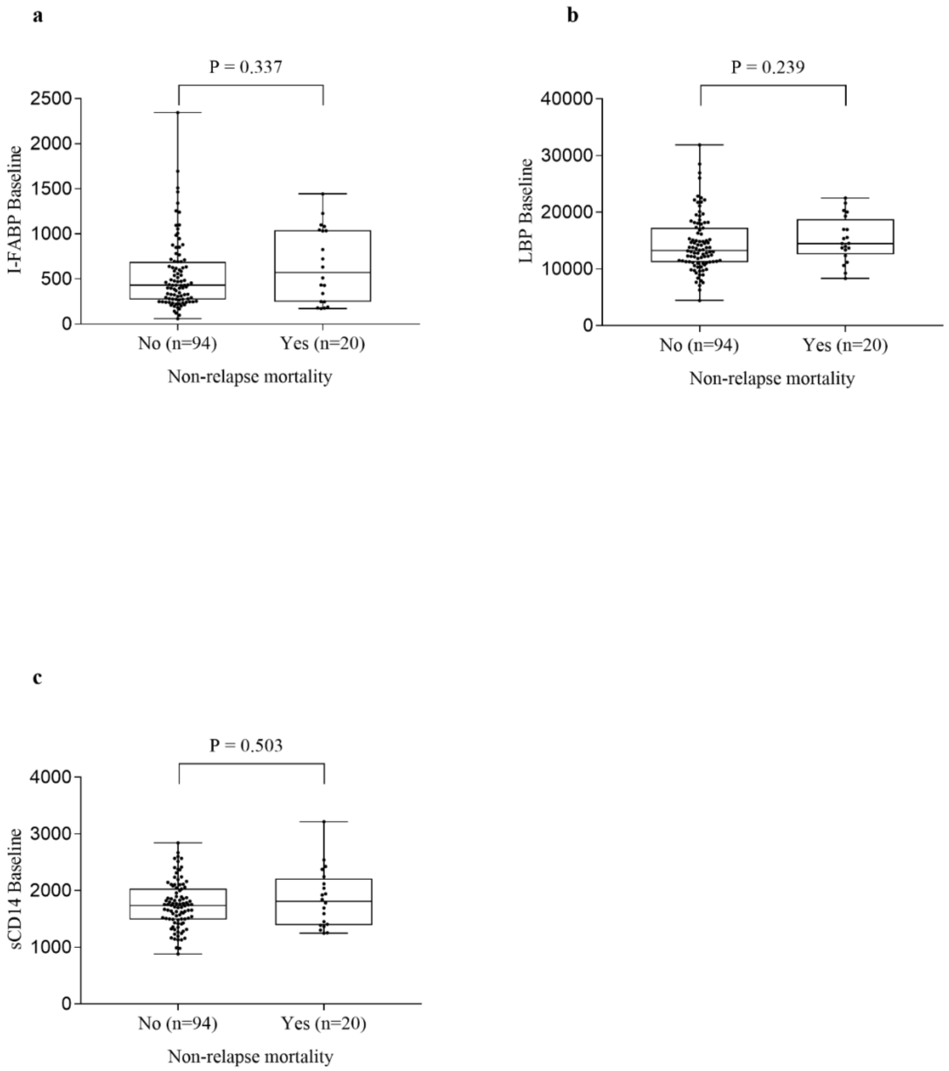

Figure 14. The relationship between gut leakage markers at 3 weeks and non-relapse mortality (NRM). **a** I-FABP and NRM, **b** LBP and NRM, **c** sCD14 and NRM. Data are I-FABP, LBP and sCD14 given as individual values (dots) and as box plots (median and interquartile range) with minimum and maximum values. P values derived from Mann-Whitney U test.

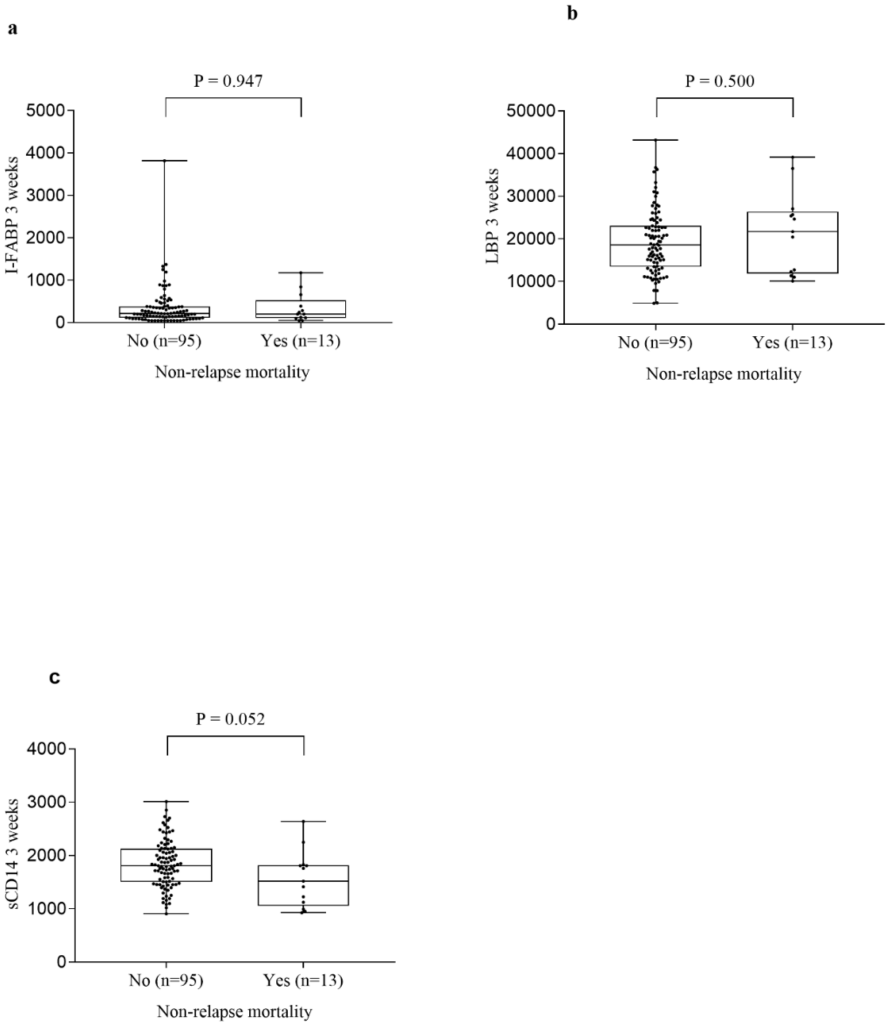

Figure 15. The relationship between markers of gut barrier functions at baseline and aGVHD. **a** I-FABP and aGVHD, **b** LBP and aGVHD, sCD14 and aGVHD. Data are I-FABP, LBP and sCD14 given as individual values (dots) and as box plots (median and interquartile range) with minimum and maximum values. P values derived from Mann-Whitney U test.

a

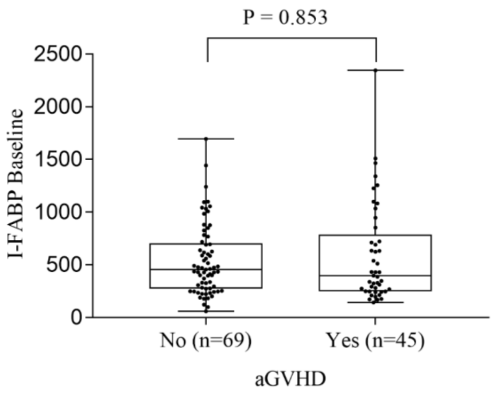

b

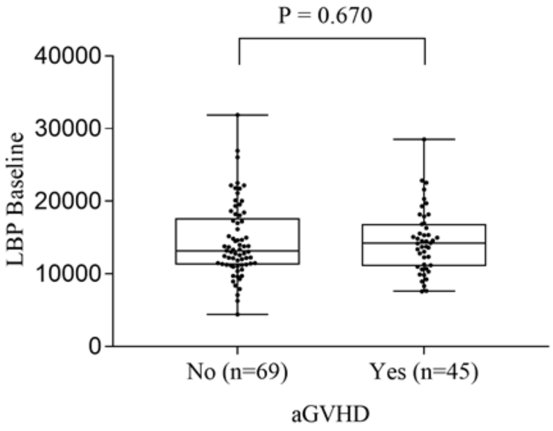

c

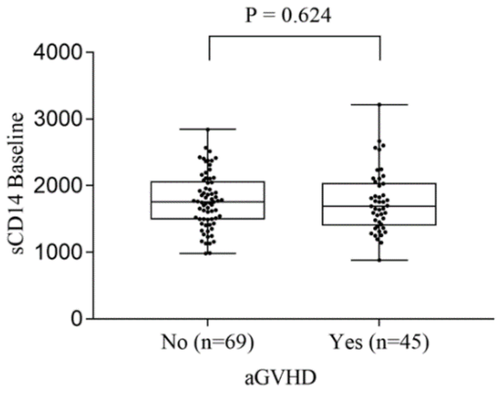

Figure 16. The relationship between gut leakage markers at 3 weeks and aGVHD. **a** I-FABP and aGVHD, **b** LBP and aGVHD, sCD14 and aGVHD. Data are IFABP, LBP and sCD14 given as individual values (dots) and as box plots (median and interquartile range) with minimum and maximum values. P values derived from Mann-Whitney U test.

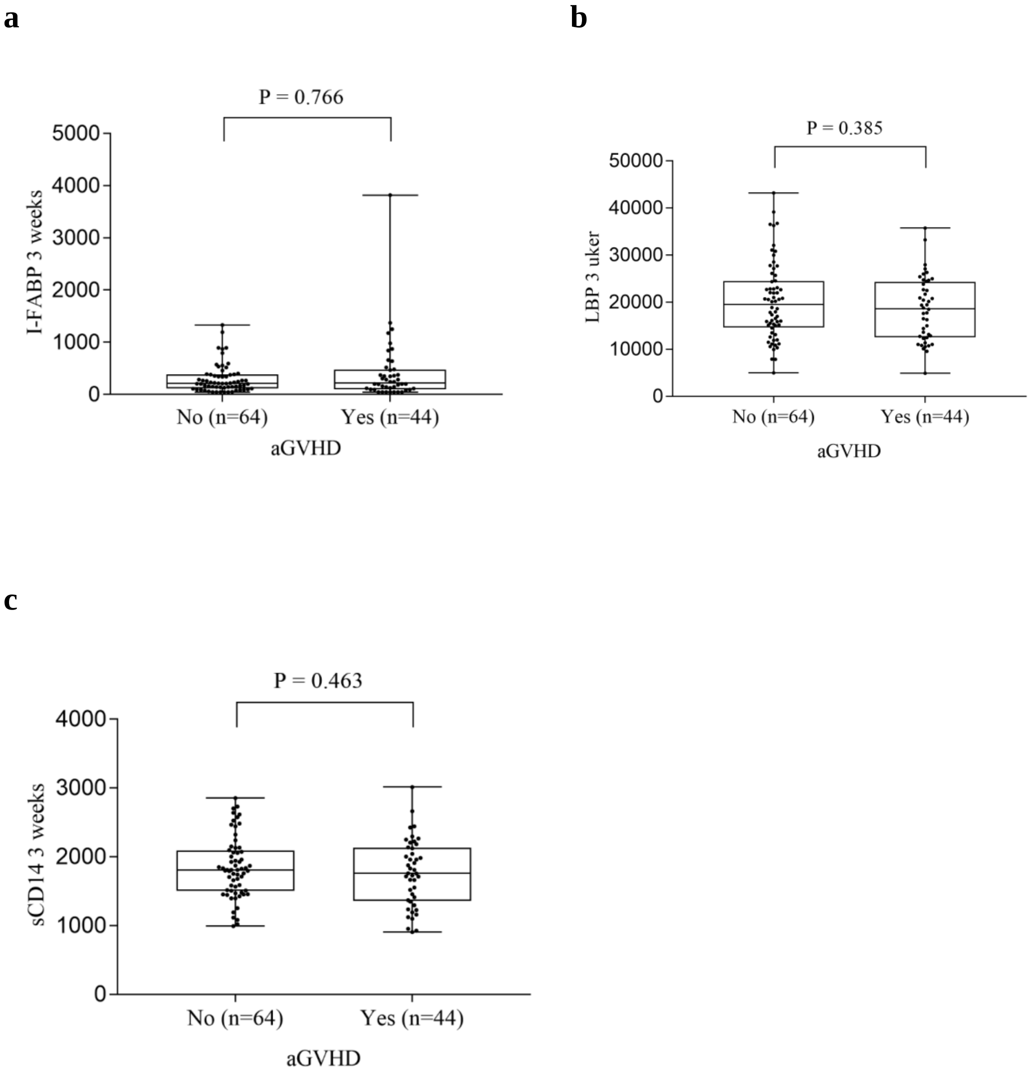

Fig. S17. The relationship between microbial diversity at baseline and HCTI-CI. **a** Observed OTUs and HCTI-CI, **b** Shannon diversity index and HCTI-CI, **c** *Blautia* abundance and HCTI-CI, **d** *Enterococcus* abundance and aGVHD. Data are observed OTUs, Shannon diversity index, *Blautia* abundance and *Enterococcus* abundance given as individual values (dots) and as box plots (median and interquartile range) with minimum and maximum values. P values derived from Mann-Whitney U test.

a

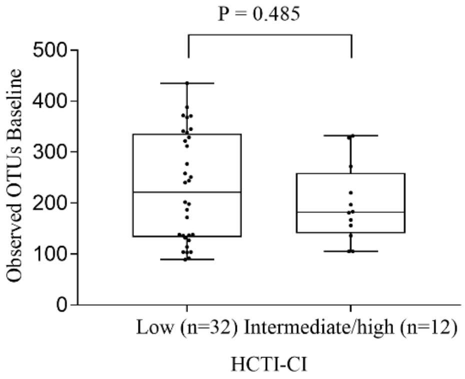

b

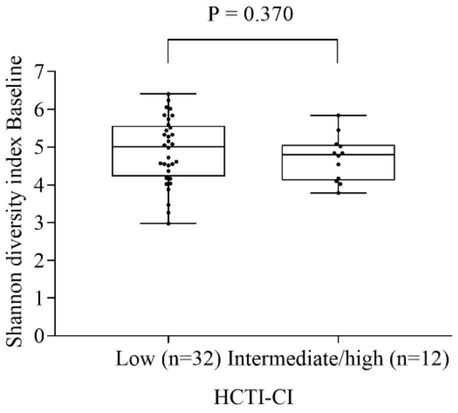

c

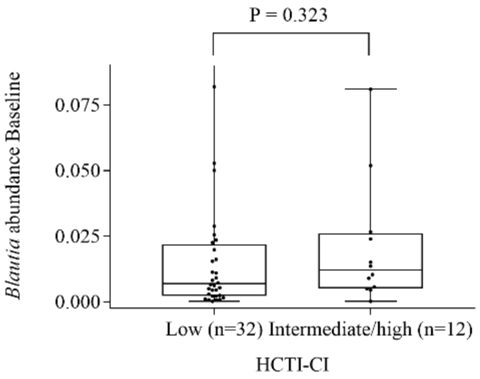

d

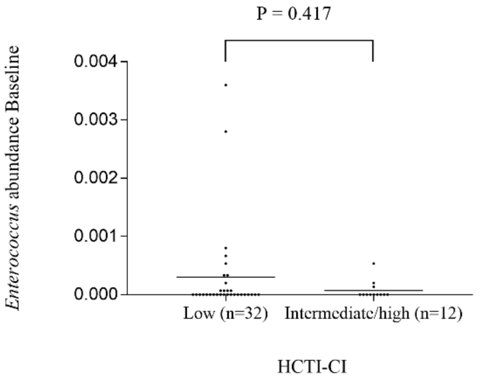

Figure S18. The relationship between microbial diversity at 3 weeks and HCTI-CI. **a** Observed OTUs and HCTI-CI, **b** Shannon diversity index and HCTI-CI, **c** *Blautia* abundance and HCTI-CI, **d** *Enterococcus* abundance and HCTI-CI. Data are observed OTUs, Shannon diversity index, *Blautia* abundance and *Enterococcus* abundance given as individual values (dots) and as box plots (median and interquartile range) with minimum and maximum values. P values derived from Mann-Whitney U test.

a

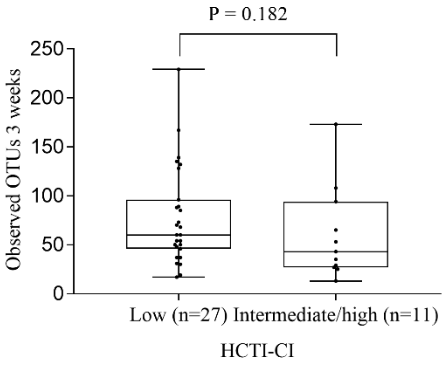

b

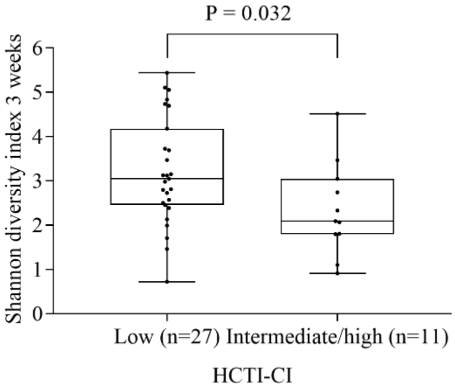

c

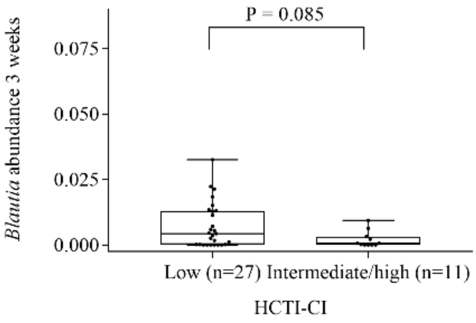

d

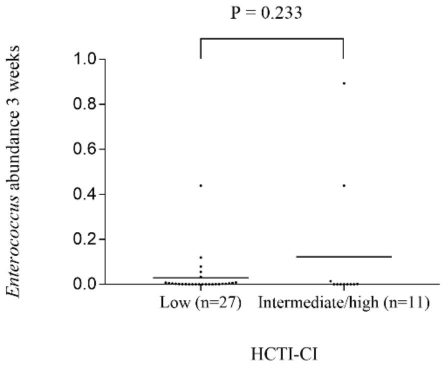

Figure S19. The relationship between SCFAs at baseline and HCTI-CI. **a** Acetic acid and HCTI-CI, **b** Propionic acid and HCTI-CI, **c** Iso-butyric acid and HCTI-CI, **d** Butyric acid and HCTI-CI, **e** Iso-valeric acid and HCTI-CI, **f** Valeric acid and HCTI-CI, **g** total SCFAs and HCTI-CI. Data are Acetic acid, Propionic acid, Iso-butyric acid, Butyric acid, Iso-valeric acid, Valeric acid and total SCFAs given as individual values (dots) and as box plots (median and interquartile range) with minimum and maximum values. P values derived from Mann-Whitney U test.

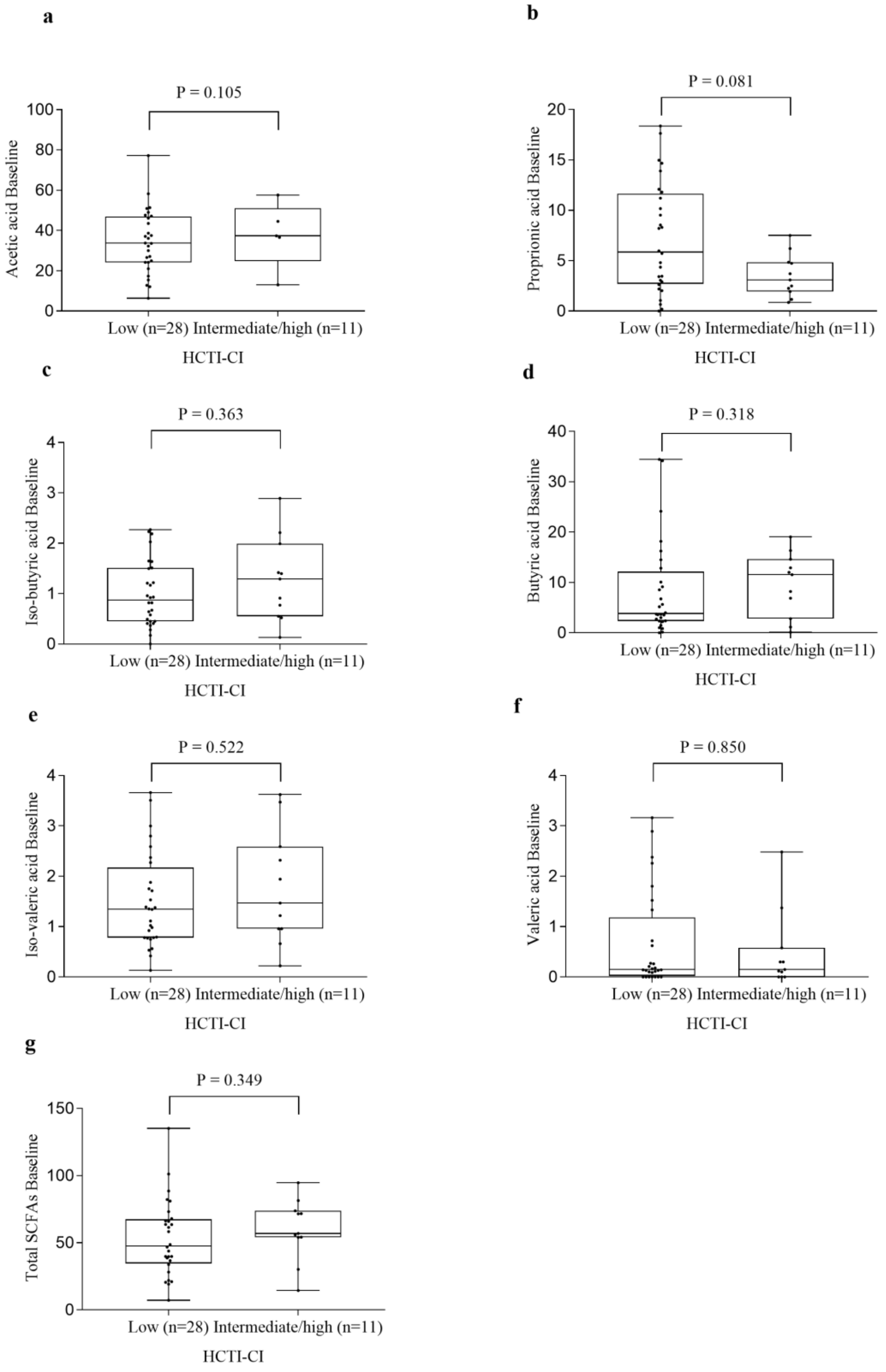

Figure S20. The relationship between markers of gut barrier functions at baseline and HCTI-CI. **a** I-FABP and HCTI-CI, **b** LBP and HCTI-CI, **c** sCD14 and HCTI-CI. Data are I-FABP, LBP and sCD14 given as individual values (dots) and as box plots (median and interquartile range) with minimum and maximum values. P values derived from Mann-Whitney U test.

a

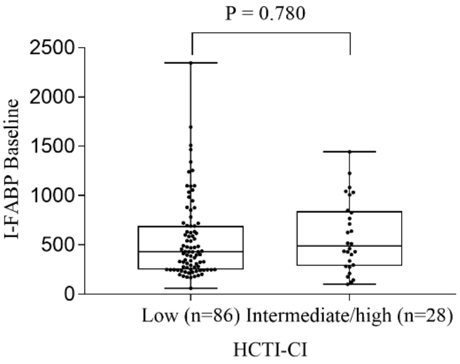

b

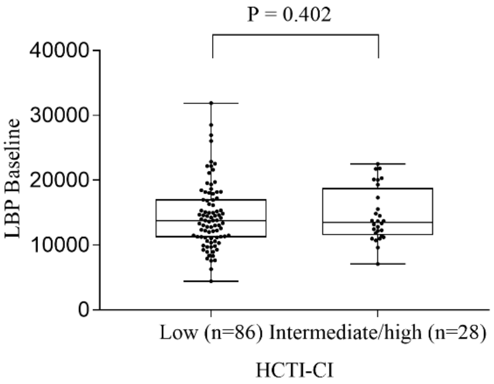

c

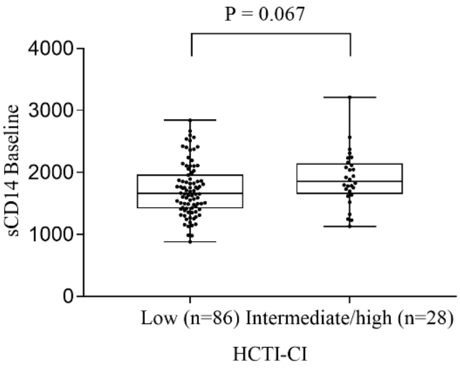

Figure S21. The relationship between gut leakage markers at 3 weeks and HCTI-CI. **a** I-FABP and HCTI-CI, **b** LBP and HCTI-CI, **c** sCD14 and HCTI-CI. Data are I-FABP, LBP and sCD14 given as individual values (dots) and as box plots (median and interquartile range) with minimum and maximum values. P values derived from Mann-Whitney U test.

a

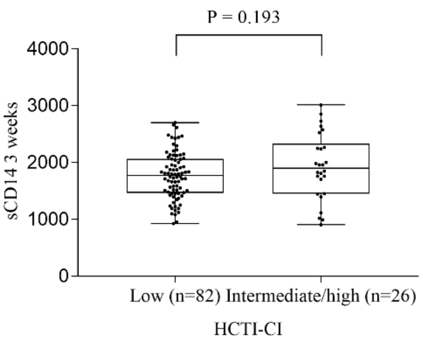

b

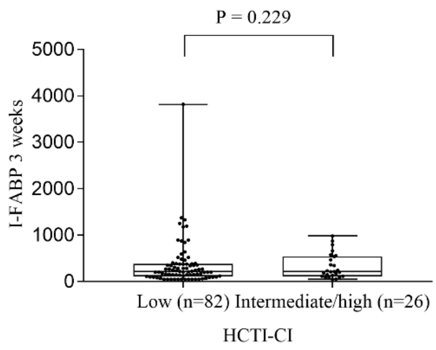

c

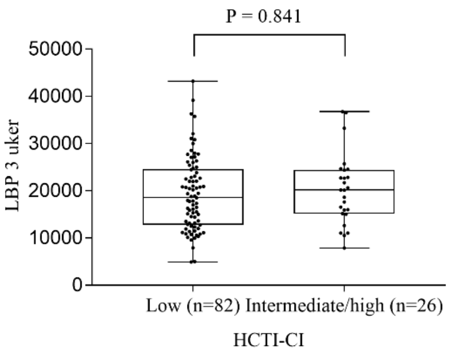

References

1 Girardot, C., Scholtalbers, J., Sauer, S., Su, S. Y. & Furlong, E. E. Je, a versatile suite to handle multiplexed NGS libraries with unique molecular identifiers. *BMC Bioinformatics* **17**, 419, doi:10.1186/s12859-016-1284-2 (2016).

2 Martin, M. Cutadapt removes adapter sequences from high-throughput sequencing reads. *EMBnet. journal* **17**, 10-12 (2011).

3 Bushnell, B., Rood, J. & Singer, E. BBMerge - Accurate paired shotgun read merging via overlap. *PLoS One* **12**, e0185056, doi:10.1371/journal.pone.0185056 (2017).

4 Caporaso, J. G. *et al.* QIIME allows analysis of high-throughput community sequencing data. *Nat Methods* **7**, 335-336, doi:10.1038/nmeth.f.303 (2010).

5 Kopylova, E., Noe, L. & Touzet, H. SortMeRNA: fast and accurate filtering of ribosomal RNAs in metatranscriptomic data. *Bioinformatics* **28**, 3211-3217, doi:10.1093/bioinformatics/bts611 (2012).

6 Bokulich, N. A. *et al.* Quality-filtering vastly improves diversity estimates from Illumina amplicon sequencing. *Nat Methods* **10**, 57-59, doi:10.1038/nmeth.2276 (2013).
